# Modeling statin myopathy in a human skeletal muscle microphysiological system

**DOI:** 10.1371/journal.pone.0242422

**Published:** 2020-11-25

**Authors:** Anandita Ananthakumar, Yiling Liu, Cristina E. Fernandez, George A. Truskey, Deepak Voora

**Affiliations:** 1 Department of Biomedical Engineering, Duke University, Durham, NC, United States of America; 2 Duke Center for Applied Genomics & Precision Medicine, Durham, NC, United States of America; UAB School of Medicine, UNITED STATES

## Abstract

Statins are used to lower cholesterol and prevent cardiovascular disease. Musculoskeletal side effects known as statin associated musculoskeletal symptoms (SAMS), are reported in up to 10% of statin users, necessitating statin therapy interruption and increasing cardiovascular disease risk. We tested the hypothesis that, when exposed to statins *ex vivo*, engineered human skeletal myobundles derived from individuals with (n = 10) or without (n = 14) SAMS and elevated creatine-kinase levels exhibit statin-dependent muscle defects. Myoblasts were derived from muscle biopsies of individuals (median age range of 62–64) with hyperlipidemia with (n = 10) or without (n = 14) SAMS. Myobundles formed from myoblasts were cultured with growth media for 4 days, low amino acid differentiation media for 4 days, then dosed with 0 and 5μM of statins for 5 days. Tetanus forces were subsequently measured. To model the change of tetanus forces among clinical covariates, a mixed effect model with fixed effects being donor type, statin concentration, statin type and their two way interactions (donor type*statin concentration and donor type* statin type) and the random effect being subject ID was applied. The results indicate that statin exposure significantly contributed to decrease in force (P<0.001) and the variability in data (R^2^C [R square conditional] = 0.62). We found no significant differences in force between myobundles from patients with/without SAMS, many of whom had chronic diseases. Immunofluorescence quantification revealed a positive correlation between the number of straited muscle fibers and tetanus force (R^2^ = 0.81,P = 0.015) and negative correlation between number of fragmented muscle fibers and tetanus force (R^2^ = 0.482,P = 0.051) with no differences between donors with or without SAMS. There is also a correlation between statin exposure and presence of striated fibers (R^2^ = 0.833, P = 0.047). In patient-derived myobundles, statin exposure results in myotoxicity disrupting SAA organization and reducing force. We were unable to identify differences in *ex vivo* statin myotoxicity in this system. The results suggest that it is unlikely that there is inherent susceptibility to or persistent effects of statin myopathy using patient-derived myobundles.

## Introduction

Statins are selective 3-hydroxy-3-methylglutaryl coenzyme A (HMG-CoA) reductase inhibitors and are among the most commonly used medications to lower cholesterol and prevent cardiovascular diseases [1]. Although randomized controlled trials demonstrate that statins are safe to use, statin associated musculoskeletal symptoms (SAMS) in the form of myopathy, myalgia, myositis, or rhabdomyolysis have been reported [2]. Myalgias typically refer to symptoms (pain, tenderness, or weakness) without associated elevations in serum creatine kinase (CK) during statin use and are reported to occur at frequencies ranging from 1% to 10% [3]. Myopathy typically refers to myalgias associated with elevation in serum CK level and occurs at a lower frequency (<5%) [4], with CK elevations greater than 10 times the upper limit of normal occurring rarely (< 0.1%) [2]. The excess of SAMS (particularly myalgia) reported in clinical practice (up to 10%) compared to blinded placebo-controlled trials is not well explained and has led many to question the causal role of statins in these cases. While there is limited insight into why certain patients exhibit myalgia vs. myopathy, the consensus (based on genetic association studies) is that SAMS is a continuum with rhabdomyolysis and myopathy representing more severe manifestations of SAMS than myalgia. Regardless, SAMS statin-induced side effects are frequently cited as a reason why many patients discontinue statin therapy [5, 6]. While many patients eventually restart statin therapy, often with the same statin type or dose; however, 1 in 3 will never restart. Consequently, SAMS is associated with higher cardiovascular morbidity [7]. Therefore, studying and addressing the biological basis of SAMS is likely to translate into improvements in population cardiovascular health.

Some of the known risk factors for SAMS are sex, dosage, hypothyroidism, statin type and advanced age [8]. Prior genetic association studies identified a common genetic variant in the *SLCO1B1* gene encoding a hepatic drug transporter that is strongly associated with an increased risk of simvastatin-induced myopathy [9] and a modest association with myalgia [10], both due to higher circulating simvastatin concentrations. Aside from underlying skeletal myopathies that can be rarely exposed during statin treatment, intrinsic features of skeletal muscle predisposing to SAMS are not known.

We have recently developed a novel microphysiological system for studying skeletal muscle *ex vivo*. This “muscle on a chip” platform uses patient-derived myoblasts to form engineered myobundles to recapitulate the organization and function of native skeletal muscle and has been applied to study the effects of statins on muscle physiology [11]. The purpose of this pilot study was to utilize this tissue engineered skeletal muscle system to determine the extent to which the clinical syndrome of SAMS is manifested in an *ex vivo* experimental model where we have already established the myotoxic effects of statins from healthy donors [11]. To accomplish this and to overcome differences in demographics and comorbidities inherent with the use of healthy donors as controls, we chose to compare cases with SAMS and elevated CK levels compared to age, sex, and statin type matched SAMS-free controls. We tested the hypothesis that patients with a history of statin induced myopathy would exhibit statin-dependent defects in muscle force and architecture when exposed to statins *ex vivo*.

## Materials and methods

### Human subjects

Using a Duke University Medical Center (DUMC) Institutional Review Board approved protocol, we used DEDUCE (Duke Enterprise Data Unified Content Explorer) a web-based portal to search DUMC patient medical records to identify potential cases and controls for this study, between June and October 2016. We used the following criteria to identify potential cases: CK value > 300 and any of the following: ICD codes (S1 Table) 2. Prescription for PCSK9 inhibitor (alurocumab or evolocumab) 3. problems listed in the “Problem List” field (S2 Table) or 4. documentation of any statin medication listed in the “Allergy” field (S2 Table). All study participants provided written informed consent.

Potential control individuals were identified as patients with an active prescription for high dosage statin, absence of any of the above case criteria from the same providers, and absence of prescription for exclusionary medications (anticoagulants and platelet P2Y12 inhibitors). This search yielded 1072 patients who subsequently underwent chart review by a single chart reviewer to confirm the presence/absence of case criteria, their dates, and provider notes in proximity to these events to search for provider documentation of attribution of findings to statins, documentation of resolution of symptoms/findings upon statin discontinuation, alternative explanations for findings, comorbidities and concomitant medications. For each case, a matched control was obtained based on statin type that was the most likely to cause the myopathy findings, sex, and current age (± 2.5 years if possible). Patients were subsequently categorized into definite case, potential cases, definite control, potential control, or none of the above. Definite cases (n = 58) controls (n = 79) that met the inclusion/exclusion criteria were approached with an introductory, IRB approved letter. Interested participants were invited for an in-person visit for informed consent, phlebotomy for a one-time biorepository blood collection for future research (plasma, DNA, RNA, and whole blood for lymphoblastoid or induced pluripotent stem cells), survey for demographics, statin and medical history, and a skeletal muscle biopsy. 42 patients were enrolled into the study (22 controls, 19 cases, and 1 was withdrawn prior to biopsy).

Key inclusion criteria common to cases/controls included: age>18 and current DUMC patient. Case-specific inclusion criteria were history of SAMS and elevated CK. Control-specific inclusion criteria were currently prescribed, tolerating, and compliant with high dose statin therapy. Key exclusion criteria for cases/controls were alternative explanation for statin myopathy or CK elevation (e.g. trauma, infection, myositis, myocardial infarction, surgery, Anti-HMG CoA reductase immune myopathy, etc.), based on chart review; use of concomitant drugs that increase risk of bleeding and cannot safely be held (warfarin, rivaroxaban, endoxaban, apixaban, dabigatran, clopidogrel, prasgurel, ticagrelor).

### Muscle biopsy procedure

A standard muscle biopsy from the *Vastus Lateralis* using previously described methods [12] was performed in each participant. Briefly, after local anesthesia (xylocaine, 2%) is injected, a small incision was made in the lateral thigh. Four to six small pieces of muscle about the size of a pea are surgically removed. The total mass of the biopsy tissue was ~ 300mg. The incision site is then closed using steri-strips.

### Culture and formation of human tissue engineered myobundles

#### Preparation and culture of human myoblasts

Human skeletal muscle samples were obtained through biopsy of *Vastus Lateralis* from 19 patients with and 22 patients without a history of statin induced myopathy. Myoblasts were isolated, expanded, cryopreserved, and passaged similar to methods described previously [11]. Briefly, the muscle samples were minced, washed in PBS with 2x Antibiotic-Antimycotic solution (Thermo Fisher), and enzymatically digested in 0.05% trypsin for 30 min. Trypsin was neutralized with hSkM (human skeletal muscle) growth media (1:1 ratio) that has low glucose (LG; 1g/L glucose) DMEM (Gibco Life Technologies) supplemented with 10% fetal bovine serum characterized (Hyclone), 0.4μg/mL dexamethasone (Sigma), 10ng/mL EGF (VWR), 50μg/mL Fetuin (Sigma), 0.1% Gentamycin (1X) (Gibco), 0.1% Amphotericin B (1X) (Gibco). Muscle was collected after centrifugation, pre-plated for 2 hours and then transferred to a growth factor reduced Matrigel (Corning) coated T-75 flask for attachment. Cells were expanded up to passage 3 and cryopreserved in 90% hSkM growth media and 10% DMSO.

#### Creation of human tissue-engineered myobundles

Cryopreserved myoblasts were thawed at passage 3 and cultured in T-175 flasks. They were passaged using 0.025% trypsin and then encapsulated in a Matrigel/fibrin matrix. The matrix contained a cell solution of 1.0 x10^6^ cells in 23 μl of 3D hSkM growth media and 2μl of 50 unit/mL thrombin in 0.1%BSA in PBS. This was added to a solution of 5 μl of 3D hSkM growth media, 10 μl of Matrigel and 10μl of 25mg/ml Fibrinogen in warm PBS. This mixture was pipetted onto the PDMS (Sylgard 184 PDMS kit, Ellsworth Adhesives, 10:1 ratio of base to curing agent) molds and anchored to Cerex frames. They were left inside the incubator for 30 mins to allow fibrin to gel. Myobundles were fed with 3ml of 3D hSkM growth media (2D hSkM growth media supplemented with 1.5 mg/ml 6-Aminocarpoic Acid (ACA)) to minimize degradation of the extracellular matrix of myobundles.

#### Culture protocol

The myobundles were cultured in 3D hSkM growth media for 4 days and shifted to a custom low amino acid (LAA) media for 4 days of differentiation. The custom LAA media contains basal media (8.3 g/ml of MEM powder media (Gibco, catalog 15230), and 2.2g/ml of sodium bicarbonate (Sigma) in 1000 ml of distilled water (Gibco)) at pH 7.2. LAA media contains 93.76% (v/v) of basal media, 2% Horse Serum, 0.666% BSA from 7.5% stock, 0.025 mg/ml Gentamycin, 0.125 μg/ml Amphotericin B from 250 μg/ml stock (Invitrogen), 250 μL 200 mM L-Carnitine (Sigma), 100 μM Fatty Acids (50 μM Oleate/50 μM Palmitate) conjugated to 0.14% BSA from 7% BSA/5mM FA stock, 5.5 mM D-Glucose from 45% stock (Sigma), 0.2 mM Pyruvate from 100 mM stock (Sigma), 2 mM Glutamine from 200 mM stock (Gibco), 1.72 μM insulin (Lonza) and 2 mg/ mL ACA [13]. The myobundles were cultured in LAA media supplemented with statins at 0 (Vehicle Control) and 5μM (High Dose) of statin in LAA for 5 days and then force tested on day 6 of statin dose. The range of concentrations were determined through literature review of human statin exposure studies of the statins of interest (Simvastatin, Atorvastatin, Lovastatin, Pravastatin and Rosuvastatin) and across a range of prescribed dosages. Based on published, plasma statin concentrations for the highest prescribed statin doses, we found that statin plasma concentrations ranged from 87–182 nM (median 140 nM) for the various statins of interest in this study [14–25]. In our pilot studies, we found that statin concentrations in the micromolar range, however, were required to produce myotoxicity in our model. Because the published range of statin concentrations was relatively narrow and to normalize exposure across patients, we chose a single statin concentration (5 μM) to expose myobundles to normalize exposure across patients.

#### Measurement of contractile force of the myobundles

The amplitude of induced contractile force by electrical or chemical stimulation is often used to evaluate the function of skeletal muscle *in vivo* and *in vitro* [11].Twitch contraction is a single contraction of the muscle at 1 Hz stimulation, whereas a tetanus contraction is a sustained muscle contraction that is brought about during the emission of action potentials at a very high rate [26]. In these studies, 20 Hz stimulation was used to induce tetanus. The testing was blinded with each pair containing one control donor and one donor that developed statin myopathy, but the identity was not known during testing.

Contractile force generation in myobundles was measured using a custom force measurement set-up [11]. Myobundles on the Cerex frame were placed between two carbon electrodes in a small 6 ml polycarbonate bath filled with LAA media at 37C. One end of the bundle was pinned to an immobile PDMS block and the other end was pinned to a floating PDMS block connected to the force transducer. Both sides of the frame were cut in order to allow for movement of the bundle during the testing. The force transducer was mounted on a motorized linear actuator so that the bundles can be stretched before electrical stimulation.

Myobundles were first stretched to 115% of their length using the linear actuator and the APT (Automatically Programmed Tool) controller user program. After stretching for 45 seconds, the twitch force of the bundle was measured by applying a single 5 millisecond rectangular pulse. The bundle was stimulated at 100V with a 1 Hz monophasic wave. Tetanus was measured by applying continuous pulses to the bundle at 100 V pulses with 20 Hz monophasic waves.

#### Immunofluorescence

Myobundles were fixed overnight using 2% paraformaldehyde (Alfa Aesar). They were then washed 3x with PBS++ (Gibco). Bundles were permeabilized overnight on a rocker in 4C using 500 μl of blocking buffer (0.2% Triton X (Sigma Aldrich), 10% goat serum (Sigma Aldrich) + 3% bovine serum albumin (Sigma Aldrich). Blocking buffer was then removed and primary antibodies, mouse sarcomeric alpha actinin (SAA Abcam ab9465 1:200) or rabbit vimentin (Abcam ab93547 1:200), were diluted in blocking buffer and 350 μl was added to each bundle and rocked for 24 hours at 4C. After 24 hours, the bundles were washed 3x with PBS++. Secondary antibodies, goat anti-mouse (ThermoFisher A11029 1:250) or goat anti-rabbit (ThermoFisher A11012) Hoechst (1:1000 ThermoFisher 33342), were added to the bundles and the bundles rocked overnight at 4C. The bundles were then washed with PBS++ 3x and imaged.

A blinded analysis of all immunofluorescence images was performed in order to quantify the number of striated and fragmented fibers controlling for their nuclei to test the hypothesis that these features are correlated with force production. The images were scored using ImageJ software. For each donor and condition, 3 images were used, a total number of nuclei per image and the number of nuclei in each striated fiber were counted and averaged to obtain “Number of nuclei in striated fibers/total number of nuclei” in the image. This provides information about the contribution of striated muscle fibers. Since each straited fiber may have more than 1 nuclei, it is important to account for the fraction of nuclei rather than the number of straited fibers. In each of the images, if fragmented fibers were present, the nuclei in each of those fragments were counted as well as the number of fragments and averaged to obtain “Number of fragmented fibers/number of nuclei in the fragmented fibers”. This metric provides information about the contribution of the fragmented fibers and as some fragmented fibers may not have any nuclei, we used the above metric rather than “Number of nuclei in fragmented fibers/total number of nuclei”.

#### Statistical analysis of force and immunoscoring data

**Fig 1:**
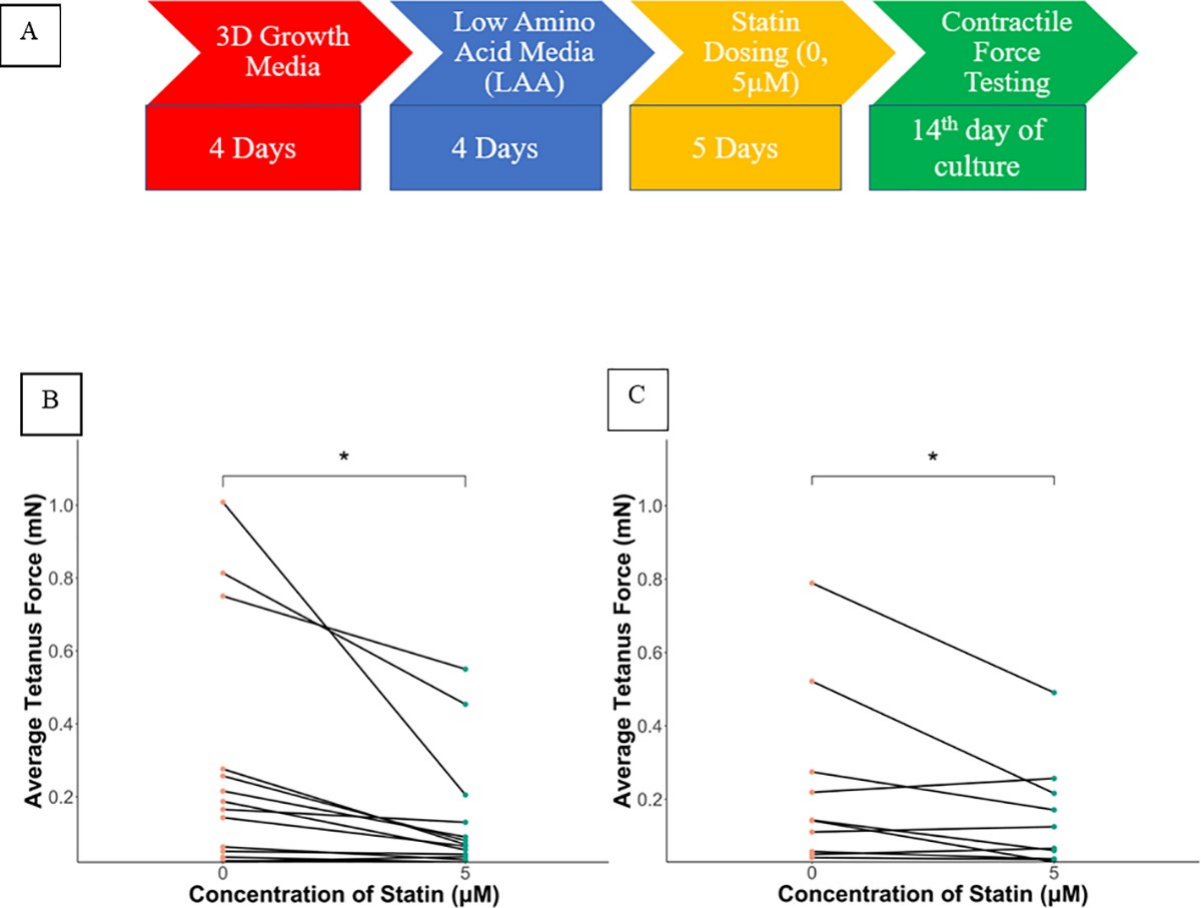
A) Culture Protocol: B) Average tetanus force of all control donors. Significant difference seen between 0 (VC) and 5μM of statin concentration. * = P = 0.012 {Data are reported as mean ±SEM, n = 3–4 biological replicates per condition with 14 control donors} C) Average tetanus force of all case donors. Significant difference is seen between 0(VC) and 5μM of statin concentration. * = P = 0.040 {Data are reported as mean ±SEM, n = 3–4 biological replicates per condition with 12 case donors}.

**Fig 2:**
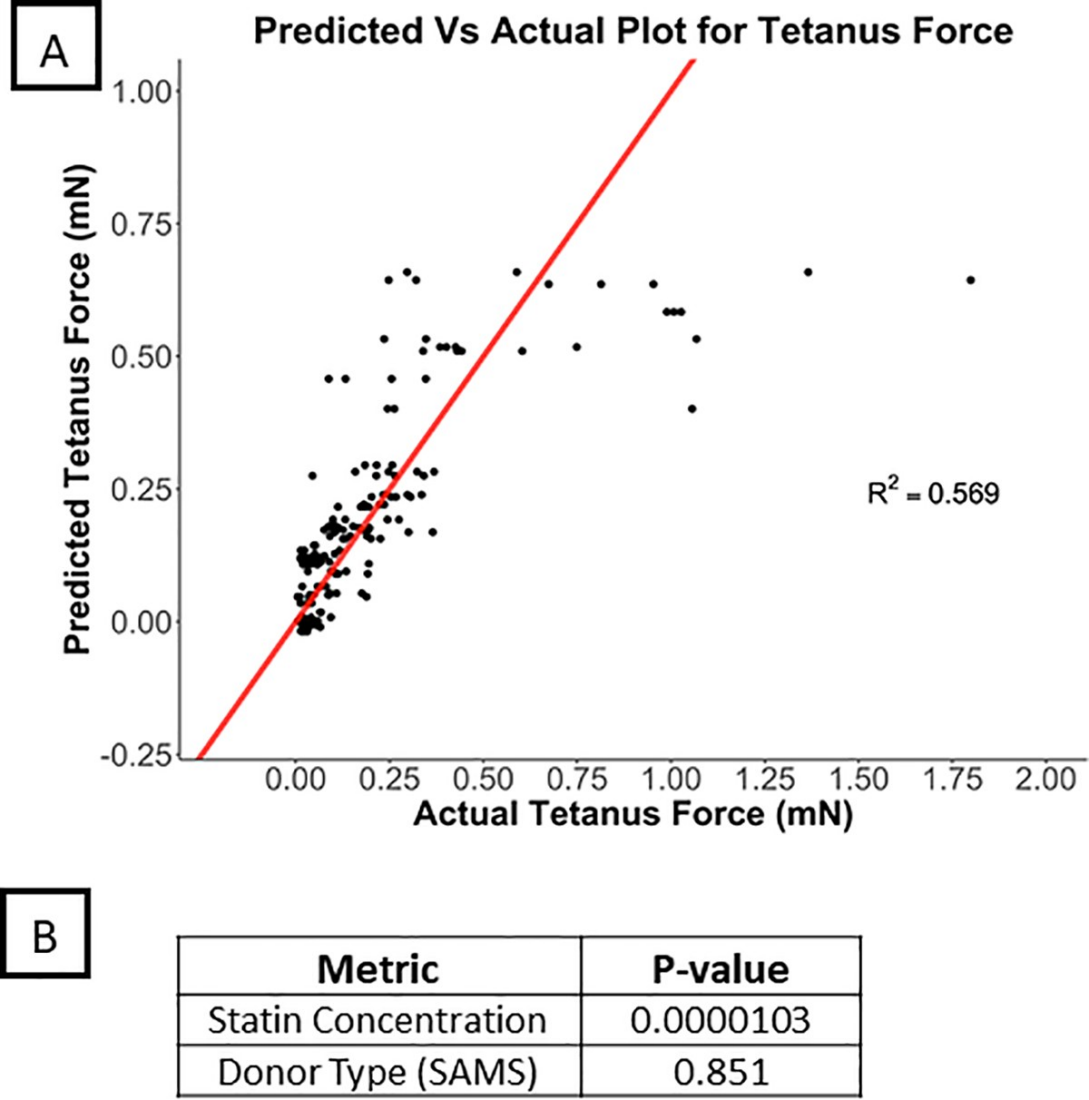
A) Predicted average tetanus values vs actual values for average of tetanus values from all donors in protocol II. B) P values of the individual and interaction effects.

Figs 1, 2 and 4 were analyzed using a mixed effect model to account for multiple measurements from the same donor [27, 28]. In Fig 1, the fixed effect was statin concentration, and the random effect was subject ID. In Fig 2, the fixed effects were donor type and statin concentration, and the random effect was subject ID. In Fig 4A, the fixed effect was tetanus force, and the random effect was subject ID. In Fig 4B, the fixed effect was tetanus force, and the random effect was subject ID. The R^2^ statistic were calculated through r.squaredGLMM function in MuMIn package [29]. A conditional R^2^ statistic was reported to measure fitness of the model. One-way ANOVA, Tukey’s HSD post hoc tests and T-tests were used to analyze the tetanus force data of young healthy donors. Detailed reports of statistical data are provided in S8 and S9 Tables.

## Results

### Patient characteristics

The baseline demographics of all enrolled participants are listed in S3 Table. The overall cohort was predominantly male, with an excess of alcohol use at the time of myopathy (75% vs. 45% for controls, p <0.01) and non-white race (50% vs. 18%, p <0.01) in cases vs. controls. The patients were recruited from the same clinics and had expected chronic conditions for patients with statin myopathy. Both cases and controls had chronic conditions and therefore would be equally affected by them. The baseline comorbidities and concomitant medications are in S4 Table. There were three cases with a potential alternative diagnosis that may have contributed to the findings of statin myopathy (117, 119, 134) that were retained in all subsequent analyses.

In terms of statin use at the time of muscle biopsy, 15 of 20 (75%) of cases were no longer taking statins due to their history of myopathy. Among the cohort studied, the most common statins reported to have caused SAMS were simvastatin and atorvastatin. The most common symptoms recorded in the electronic health records were pain, weakness, and stiffness and the statin most likely to have caused myopathy in cases are in S5 Table. Controls, who were individuals who tolerated statins, were prescribed atorvastatin (11/22), simvastatin (5/22), rosuvastatin (4/22), and pravastatin (2/22). We surveyed cases and controls for symptoms that could be attributed to statins and found that while they were more common in cases, as expected based on their non-specific nature, they were not absent in controls (S6 Table). The average weight of the tissue obtained by muscle biopsy was 284 mg with no differences between cases/controls. There were no adverse events related to biopsy procedure.

### Contractile force measurement

To establish conditions under which the muscle responded to a range of lovastatin concentrations (0.0, 2 μM, 3 μM, 5 μM), myobundles were fabricated using myoblasts isolated from healthy young donors (N = 3–4), cultured in LAA differentiation media for 5 days, and exposed to lovastatin for 5 or 10 days. Significant effects of 5μM lovastatin exposure on force was seen (P<0.05) (S1A Fig). There was a significant reduction in force the longer the myobundles were cultured (~68% reduction in force in control bundles cultured for 10 vs 5 days) (S1B Fig). A plot of normalized average tetanus force vs dose*exposure time shows a negative relationship between force and dose*exposure which indicated that a higher dose of statin concentration can be used for a shorter period of time to assess the effect of statins (R^2^ = 0.86, p = 0.007) (S2 Fig). Based on these results, patient myobundles were tested at 5 μM statin after 5 days of exposure.

From the initial set of patients, 24 patients consisting of 10 cases and 14 controls were available to be tested for contractile force under this protocol. The remaining cases/controls did not have a sufficient number of cells to be tested under this protocol. S7 Table contains data regarding the type of statin with which they were dosed along with their gender, age, and creatinine kinase level of the case donors. Fig 1A provides the experimental design. Exposure to 5 μM statin reduced the mean contractile force for both myobundles from controls (Fig 1B) and cases (Fig 1C). There was a significantly lower force between vehicle control and 5μM of statin concentration in myobundles from control donors (R^2^ = 0.685P = 0.012) (Fig 1B). In the myobundles from cases, statin exposure also significantly lowered forces, (R^2^ = 0.812, P = 0.040) (Fig 1C).

### Statistical analysis of factors affecting contractile force

To evaluate the change in contractile force in response to statin exposure within myobundles derived from the same patient, we used a mixed effects model with the fixed effects being donor type (to compare changes in myobundles from cases vs. control) and statin concentration, and the random effect was subject ID. From this analysis, statin exposure was significantly associated with tetanus force (P < 0.001) and explained over 56% of the variability in the observed data (R^2^ = 0.569) (Fig 2). We found no differences between case and control myobundles with respect to tetanus force (P = 0.85). Further, the effect of statin exposure on tetanus force did not differ by case/control myobundles (statin concentration x myopathy status interaction (P = 0.28). Twitch kinetics and fatigue measurements also did not show a difference between case/control myobundles (S5 and S6 Figs). Therefore, within this system that is sensitive to the myotoxic effects of statins, we were unable to detect any significant differences between patients with and without a history of statin myopathy using force measurements in response to statin exposure.

### Immunofluorescence imaging and quantification

**Fig 3:**
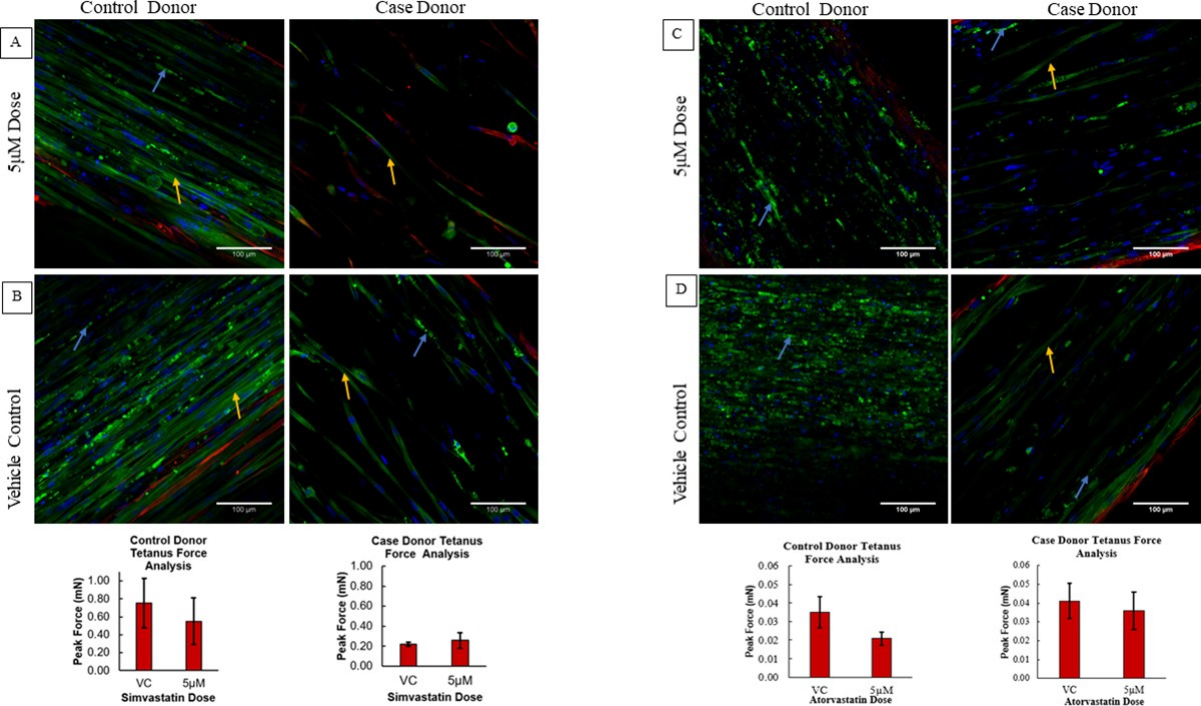
Representative immunofluorescence images of donors along with their tetanus force production. Yellow Arrows (Striated Fiber), Blue Arrows (Fragmented Fibers). A) Striated muscle fibers (control donor dosed with simvastatin) are correlated with higher force production when compared to B) Case donor dosed with simvastatin which had reduced number of straited fibers. Increased fragmented fibers are seen in C) control donor and less striated fibers are seen in D) case donor dosed with Atorvastatin which is correlated to their tetanus force production.

To explore further the effects of statin exposure and the potential mechanism by which statin exposure lowers force in this system we used 3D immunofluorescence (IF) imaging of the myobundles (19 of 24 donors) using SAA staining, which is a late maturation marker in myotubules and vimentin, which stains for fibroblasts. Representative immunofluorescence images along with their individual tetanus force measurements are seen in Fig 3. Healthy muscle bundles in our system show striated muscle fibers which are indicative of their ability to provide the architecture for adequate contractile force production (S3 Fig). For the patient-derived myobundles in this study, there were fewer cells expressing SAA and a higher number of fragmented myofibers as well as myofibers that do not express striations.

**Fig 4:**
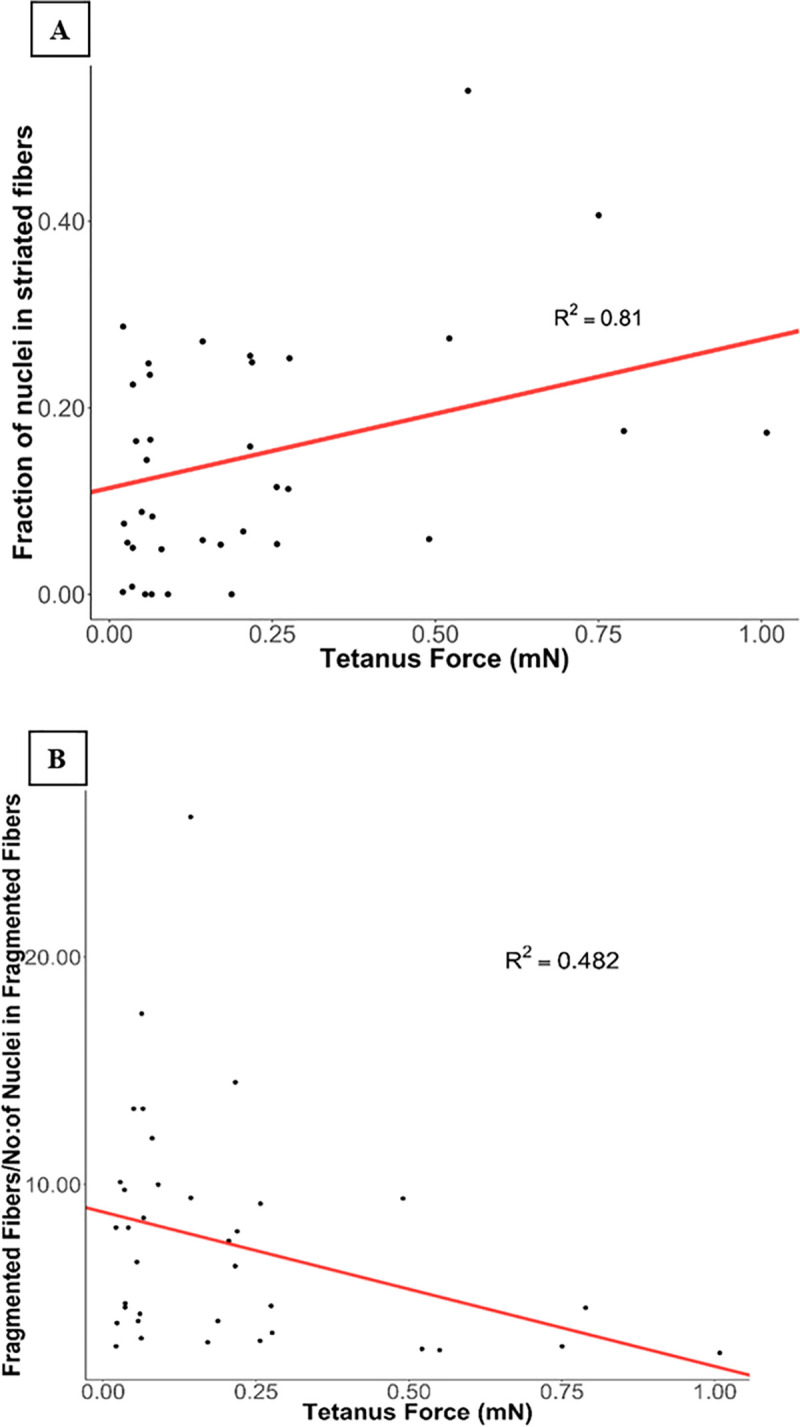
A) Positive correlation between striated fibers/total number of nuclei in image field and tetanus force (P = 0.015). B) Negative correlation between fragmented fibers/number of nuclei in fragmented fibers and tetanus force (P = 0.051).

A mixed effect model was used to determine the relationship between IF measures and tetanus force and myobundle case/control status. There was a positive correlation between the fraction of nuclei in striated fibers and tetanus force (R^2^ = 0.81; P = 0.015) (Fig 4A). Further analysis of the data showed a correlation between statin exposure and presence of striated fibers. There was a reduction in striated fibers with statin exposure (S4 Fig, R^2^ = 0.833, P = 0.047). Thus, a higher fraction of nuclei in striated muscle fibers is associated with higher tetanus force production. We also observed a trend towards negative correlation between fragmented fibers/number of nuclei in fragmented fibers and tetanus force (R^2^ = 0.482; P = 0.051) (Fig 4B). We did not find any significant association between case/control myobundles or interactions with statin concentration with either IF measures (for fragmented fibers/number of nuclei in fragmented fibers: a) between case and control (P = 0.57, R^2^ = 0.426), b) interactions of case/control and statin concentrations (P = 0.80, R^2^ = 0.83), for fraction of nuclei in striated fibers: a) between case and control(P = 0.51, R^2^ = 0.802), b) interactions of case/control and statin concentration (P = 0.75, R^2^ = 0.385).

## Discussion

Statins are a major class of medicines to lower cholesterol and prevent cardiovascular diseases. Statin therapy is associated with musculoskeletal side effects in the form of myopathy, myalgia, myositis or rhabdomyolysis which can lead to an interruption in statin treatment. A complete understanding of the exact pathology or mechanism of statin induced myopathy is lacking. Hence, better systems such as human, *ex vivo* skeletal muscle systems may yield better methods to study SAMS. We used a previously described *ex vitro* tissue-engineered skeletal muscle system [11] to model the effects of statins on skeletal muscle in patients who received statins and did not report SAMS vs. those who did report SAMS to determine the extent to which there are differences between patients with and without SAMS. In this pilot study, we chose “extreme” manifestations of SAMS for cases (defined as elevated CK) and controls (able to tolerate high-dose statins) to help detect differences. We found that statin exposure has a myotoxic effect on myobundle force generation and sarcomere structure however we found within the limits of this system, we were unable to detect differences in sensitivity to statins in myobundles derived from patients with or without SAMS in this pilot study.

Based on blinded randomized clinical trial data, statin therapy is infrequently associated with myopathy and that most with symptoms do not have myopathy [30]. This may, in part, explain why we were unable to identify significant differences between the myobundles in our study despite selecting patient donor cases with objective evidence (i.e. elevated CK levels) of myopathy. Alternatively, because cases were historical (i.e. myopathy had occurred in the past) and the case donors were no longer taking statins, our findings suggest there may be no inherent susceptibility to SAMS and that the effects do not persist after statin discontinuation. Moreover, while culturing the cells, the healthiest cells may have been selected which could have concealed myopathy in these donors or prevented a pronounced effect from showing through during force measurement. Despite our efforts at creating homogeneous groups of cases/controls through matching, residual differences in their clinical characteristics may have obscured any measurable differences in this system such as higher alcohol use in cases, controls who reported muscle pain despite being able to tolerate their statins, or the differences in statin use at the time of biopsy between cases/controls. A study also showed that statin induced myalgia was not associated with impaired capacity for oxidative phosphorylation or ROS signaling. Therefore, there was no difference in oxidative phosphorylation for myotubes of patients with muscle pain and weakness relative to controls [31] which could explain to some part why we are not seeing obvious differences in force production. Lastly, due to the limited sample size we were unable to further adjust for these additional differences in our models.

Other reasons why we were unable to detect any significant differences are; First, while this system has many features that mimic skeletal muscle and has the advantage of being patient-derived, there are limitations in extrapolating to human pathology including lower contractile force production, quicker depletion of satellite cells, lacking cell interactions with other cell types and shorter lifespan compared to native skeletal muscle. Second, in this study, cases and controls were typical of patients using statins: older age and with a wide range of clinical diseases including cardiovascular disease, cerebrovascular disease, diabetes, coronary artery disease, obesity, hypothyroidism. Consequently, the donors from the current study also produced significantly lower forces when compared to young healthy control donors from our control (S3 Fig) and prior studies [11, 32]. Further, the engineered muscle from these older donors who had conditions that made them eligible for statin therapy also had disorganized myofibers, something not observed in myofibers from young, healthy donors. Therefore, these diseases–by lowering overall force measurements—may have limited our sensitivity to detect subtle effects of statin exposure in myopathy cases for which we were not powered to detect in this pilot study. Because SAMS is a frequent cause of premature statin discontinuation and our interest in identifying “extreme” cases/controls, there was marked imbalance in statin use at the time of muscle biopsy. Although this difference in statin use may have introduced a difference in the baseline myobundle state, our interest focused on changes in muscle force in response to statin exposure. Therefore, we do not believe that differences in baseline statin use obscured our ability to detect differences between cases/controls. Lastly, this study aimed to identify the *direct* effects of statins on skeletal muscle with respect to myopathy over a relatively short period of time (5 days). The lack of difference between SAMS cases and controls may be due to indirect effects of statins on skeletal muscle (e.g., changes in circulating lipid composition) that may impact myopathy. In addition, it may be that a longer duration of statin exposure (e.g. months) may be needed to expose a statin myopathy phenotype. Future work employing multi-system microphysiological models that incorporate hepatic and skeletal muscle systems for longer durations of exposure may be better suited to explore indirect effects on statins on muscle tissue.

Although we were unable to detect differences in SAMS cases and controls, we were able to further demonstrate that statins have a myotoxic effect in our system. Our original observations were in myobundles derived from healthy volunteers. The findings in the current study demonstrate that statins have a myotoxic effects in myobundles made from donors with and without a history of SAMS. We also further our understanding of the determinants of force in our model. The immunofluorescence quantification revealed a positive correlation between the ratio of striated fibers/number of nuclei to tetanus force measurement. The primary function of striated muscle fibers is to generate force [33] and our system has been able to successfully fabricate skeletal muscle which recapitulate the key functionality and architecture of native human skeletal muscle. Therefore, the presence of fully formed striated muscle fibers results in higher contractile muscle function.

The reduction in fully formed striated muscle fibers with statin exposure in our model, in part, sheds light on the mechanism by which statins exert a myotoxic effect. While the exact mechanism of this myotoxicity is unknown, skeletal muscle express cholesterol biosynthetic pathway genes including *HMGCR* that are upregulated in the patient derived myobundle in this system during statin exposure (unpublished data). Future work examining the role of *HMGCR* in skeletal muscle fiber development and maintenance may shed further insight into the mechanism of statin myotoxicity. Ongoing work on metabolomics and RNA sequencing of the media and myobundles could help understand the characterization of myobundles and their response to statin exposure on a molecular level. This could provide more details regarding the mechanistic effect of statin and observe if there are any intrinsic factors that may explain patient’s susceptibility to statin therapy.

## Supporting information

S1 Data (ZIP)

S1 Table Diagnosis codes used to search for potential cases. (DOCX)

S2 Table Problem names used to search problem lists to identify case. (DOCX)

S3 Table Baseline donor demographics. (DOCX)

S4 Table Baseline comorbidities and concomitant medications. (DOCX)

S5 Table Myopathy symptoms in cases. (DOCX)

S6 Table Patient-reported symptoms: Answer 1 in any of the questions in muscle weakness, muscle pain, muscle stiffness. (DOCX)

S7 Table Statin type, gender, age and creatinine kinase level of donors tested. (DOCX)

S8 Table Details of statistical data including parameter, estimate, standard error, degree of freedom, t value, p-value, confidence interval. (DOCX)

S9 Table Details of statistical data including AIC, RM, RC and restricted log likelihood. (DOCX)

S1 Fig Myobundles made with myoblasts from healthy donors received (A) 2, 3 and 5 μM of lovastatin for 5 days (B) 2 μM of lovastatin for 10 days. There is a significant difference between conditions with respect to their respective vehicle controls. * p < 0.05. A ~58% reduction in force is seen between VC at day 5 and Day 10. Data reported as mean ±SEM, n = 3–4 biological replicates per condition.
(TIF)

S2 Fig Negative relationship between force and dose*exposure with young healthy donors proves that a higher dose of statin concentration can be used for a shorter period to assess the effect of statins. (TIF)

S3 Fig Striated muscle fibers seen in confocal image of young healthy donor. (TIF)

S4 Fig There is a reduction in fraction of nuclei in striated fibers with statin exposure. P<0.05.
(TIF)

S5 Fig A, B. There is no significant differences in Twitch Kinetics between case and control.
(TIF)

S6 Fig There is no significant differences in fatigue measurements between case and control. (TIF)

S7 Fig Myoblast purity between case and control donors does not show a difference. (TIF)

## References

[1] Erratum: 2013 ACC/AHA Guideline on the treatment of blood cholesterol to reduce atherosclerotic cardiovascular risk in adults: A report of the American college of cardiology/American Heart Association task force on practice guidelines (Circulation (2014) 129: Supplement 2 (S1–S45) 10.1161/01.cir.0000437738.63853.7a). Vol. 129, Circulation. Lippincott Williams and Wilkins; 2014. p. S46–8. 24222016

[2] Tomaszewski M, Stêpieñ KM, Tomaszewska J, Czuczwar SJ. Statin-induced myopathies. 2011 10.1016/s1734-1140(11)70601-6 22001973

[3] Feng Q, Wilke RA, Baye TM. Individualized risk for statin-induced myopathy: Current knowledge, emerging challenges and potential solutions. Vol. 13, Pharmacogenomics. 2012 p. 579–94. 10.2217/pgs.12.11 22462750 PMC3337775

[4] Moghadam-Kia S, Oddis C V., Aggarwal R. Approach to asymptomatic creatine kinase elevation. Cleve Clin J Med. 2016;83(1):37–42. 10.3949/ccjm.83a.14120 26760521 PMC4871266

[5] Mancini GBJ, Baker S, Bergeron J, Fitchett D, Frohlich J, Genest J, et al Diagnosis, Prevention, and Management of Statin Adverse Effects and Intolerance: Proceedings of a Canadian Working Group Consensus Conference. Vol. 27, Canadian Journal of Cardiology. 2011 p. 635–62. 10.1016/j.cjca.2011.05.007 21963058

[6] Cohen JD, Brinton EA, Ito MK, Jacobson TA. Understanding Statin Use in America and Gaps in Patient Education (USAGE): An internet-based survey of 10,138 current and former statin users. J Clin Lipidol. 2012;6(3):208–15. 10.1016/j.jacl.2012.03.003 22658145

[7] Serban MC, Colantonio LD, Manthripragada AD, Monda KL, Bittner VA, Banach M, et al Statin Intolerance and Risk of Coronary Heart Events and All-Cause Mortality Following Myocardial Infarction. J Am Coll Cardiol. 2017 3 21;69(11):1386–95. 10.1016/j.jacc.2016.12.036 28302290

[8] Jacobson TA. Toward “pain-free” statin prescribing: Clinical algorithm for diagnosis and management of myalgia. Vol. 83, Mayo Clinic Proceedings. Elsevier Ltd; 2008 p. 687–700. 10.4065/83.6.687 18533086

[9] Meade T, Sleight P, Collins R, Armitage J, Parish S, Barton J, et al SLCO1B1 variants and statin-induced myopathy—A genomewide study. N Engl J Med. 2008 8 21;359(8):789–99. 10.1056/NEJMoa0801936 18650507

[10] Voora D, Shah SH, Spasojevic I, Ali S, Reed CR, Salisbury BA, et al The SLCO1B1*5 Genetic Variant Is Associated With Statin-Induced Side Effects. J Am Coll Cardiol. 2009 10 20;54(17):1609–16. 10.1016/j.jacc.2009.04.053 19833260 PMC3417133

[11] Madden L, Juhas M, Kraus WE, Truskey GA, Bursac N. Bioengineered human myobundles mimic clinical responses of skeletal muscle to drugs. Elife. 2015 1 8;2015(4). 10.7554/eLife.04885 25575180 PMC4337710

[12] Huffman KM, Koves TR, Hubal MJ, Abouassi H, Beri N, Bateman LA, et al Metabolite signatures of exercise training in human skeletal muscle relate to mitochondrial remodelling and cardiometabolic fitness. Diabetologia. 2014 10 1;57(11):2282–95. 10.1007/s00125-014-3343-4 25091629 PMC4182127

[13] Kondash ME, Ananthakumar A, Khodabukus A, Bursac N, Truskey GA. Glucose Uptake and Insulin Response in Tissue-engineered Human Skeletal Muscle. Tissue Eng Regen Med [Internet]. 2020 3 21 [cited 2020 Oct 24];1–13. Available from: https://link.springer.com/article/10.1007/s13770-020-00242-y 32200516 10.1007/s13770-020-00242-y PMC7710786

[14] Shitara Y, Sugiyama Y. Pharmacokinetic and pharmacodynamic alterations of 3-hydroxy-3-methylglutaryl coenzyme A (HMG-CoA) reductase inhibitors: Drug-drug interactions and interindividual differences in transporter and metabolic enzyme functions. Vol. 112, Pharmacology and Therapeutics. Pergamon; 2006 p. 71–105. 10.1016/j.pharmthera.2006.03.003 16714062

[15] Kantola T, Kivistö KT, Neuvonen PJ. Grapefruit juice greatly increases serum concentrations of lovastatin and lovastatin acid*. Clin Pharmacol Ther [Internet]. 1998 4 1 [cited 2020 Oct 24];63(4):397–402. Available from: http://doi.wiley.com/10.1016/S0009-9236(98)90034-0 9585793 10.1016/S0009-9236(98)90034-0

[16] Hatanaka T. Clinical pharmacokinetics of pravastatin: Mechanisms of pharmacokinetic events [Internet]. Vol. 39, Clinical Pharmacokinetics. Adis International Ltd; 2000 [cited 2020 Oct 24]. p. 397–412. Available from: https://pubmed.ncbi.nlm.nih.gov/11192473/ 10.2165/00003088-200039060-00002 11192473

[17] Lemahieu WPD, Hermann M, Asberg A, Verbeke K, Holdaas H, Vanrenterghem Y, et al Combined therapy with atorvastatin and calcineurin inhibitors: No interactions with tacrolimus. Am J Transplant [Internet]. 2005 9 1 [cited 2020 Oct 24];5(9):2236–43. Available from: https://onlinelibrary.wiley.com/doi/full/10.1111/j.1600-6143.2005.01005.x 16095503 10.1111/j.1600-6143.2005.01005.x

[18] Martin PD, Warwick MJ, Dane AL, Hill SJ, Giles PB, Phillips PJ, et al Metabolism, Excretion, and Pharmacokinetics of Rosuvastatin in Healthy Adult Male Volunteers. Clin Ther. 2003 11 1;25(11):2822–35. 10.1016/s0149-2918(03)80336-3 14693307

[19] Simonson SG, Martin PD, Mitchell P, Schneck DW, Lasseter KC, Warwick MJ. Pharmacokinetics and pharmacodynamics of rosuvastatin in subjects with hepatic impairment. Eur J Clin Pharmacol [Internet]. 2003 2 1 [cited 2020 Oct 24];58(10):669–75. Available from: https://link.springer.com/article/10.1007/s00228-002-0541-7 12610743 10.1007/s00228-002-0541-7

[20] Keskitalo J, Kurkinen K, Neuvonen P, Niemi M. ABCB1 Haplotypes Differentially Affect the Pharmacokinetics of the Acid and Lactone Forms of Simvastatin and Atorvastatin. Clin Pharmacol Ther [Internet]. 2008 10 26 [cited 2020 Oct 24];84(4):457–61. Available from: http://doi.wiley.com/10.1038/clpt.2008.25 19238649 10.1038/clpt.2008.25

[21] Lennernäs H. Clinical Pharmacokinetics of Atorvastatin [Internet]. Vol. 42, Clinical Pharmacokinetics. Clin Pharmacokinet; 2003 [cited 2020 Oct 24]. p. 1141–60. Available from: https://pubmed.ncbi.nlm.nih.gov/14531725/ 10.2165/00003088-200342130-00005 14531725

[22] Olsson AG, McTaggart F, Raza A. Rosuvastatin: A Highly Effective New HMG-CoA Reductase Inhibitor. Cardiovasc Drug Rev [Internet]. 2006 6 7 [cited 2020 Oct 24];20(4):303–28. Available from: http://doi.wiley.com/10.1111/j.1527-3466.2002.tb00099.x 10.1111/j.1527-3466.2002.tb00099.x 12481202

[23] FDA Clinical Review. Rosuvastatin Clinical Review—FDA [Internet]. [cited 2020 Oct 24]. Available from: https://www.fda.gov/media/78285/download

[24] FDA Label. Highlights of Prescribing Information—SIMCOR [Internet]. [cited 2020 Oct 24]. Available from: https://www.accessdata.fda.gov/drugsatfda_docs/label/2012/022078s013lbl.pdf

[25] FDA Label. Highlights of Prescribing Information—Pravachol [Internet]. [cited 2020 Oct 24]. Available from: https://www.accessdata.fda.gov/drugsatfda_docs/label/2012/019898s062-trackedchangeslbl.pdf

[26] Rüegg JC. Muscle Contraction: Molecular and Cellular Physiology In: Comprehensive Human Physiology. Springer Berlin Heidelberg; 1996 p. 935–57.

[27] Bates D, Mächler M, Zurich E, Bolker BM, Walker SC. Fitting Linear Mixed-Effects Models Using lme4. [cited 2019 Oct 19]; Available from: https://www.jstatsoft.org/

[28] Satterthwaite FE. An Approximate Distribution of Estimates of Variance Components. Biometrics Bull. 1946 12;2(6):110 20287815

[29] Nakagawa S, Johnson PCD, Schielzeth H. The coefficient of determination R2 and intra-class correlation coefficient from generalized linear mixed-effects models revisited and expanded. J R Soc Interface. 2017 9 1;14(134). 10.1098/rsif.2017.0213 28904005 PMC5636267

[30] Peto R, Collins R. Trust the blinded randomized evidence that statin therapy rarely causes symptomatic side effects. Circulation. 2018;138(15):1499–501. 10.1161/CIRCULATIONAHA.118.036846 30354509

[31] Dohlmann TL, Morville T, Kuhlman AB, Chrøis KM, Helge JW, Dela F, et al Statin Treatment Decreases Mitochondrial Respiration but Muscle Coenzyme Q10 Levels Are Unaltered: The LIFESTAT Study. J Clin Endocrinol Metab [Internet]. 2019 3 21 [cited 2020 Oct 22];104(7):2501–8. Available from: https://pubmed.ncbi.nlm.nih.gov/30299473/ 10.1210/jc.2018-01185 30299473

[32] Davis BN, Yen R, Prasad V, Truskey GA. Oxygen consumption in human, tissue-engineered myobundles during basal and electrical stimulation conditions. APL Bioeng. 2019 6;3(2):026103 10.1063/1.5093417 31149650 PMC6520098

[33] Shadrin IY, Khodabukus A, Bursac N. Striated muscle function, regeneration, and repair Vol. 73, Cellular and Molecular Life Sciences. Birkhauser Verlag AG; 2016 p. 4175–202. 10.1007/s00018-016-2285-z 27271751 PMC5056123

