# Supplementary material for: Modeling statin myopathy in a human skeletal muscle microphysiological system
Source: PLoS One. 2020 Nov 25;15(11):e0242422. doi: 10.1371/journal.pone.0242422 (PMC7688150; doi:10.1371/journal.pone.0242422)
Supplement: S1 Data — (ZIP) [file pone.0242422.s001.zip › Data Repository/Scripts/codes_for_submission_main-analysis_S4.html]

Modeling Statin Myopathy in a Human Skeletal Muscle Microphysiological System - Main Analysis + S4


# Modeling Statin Myopathy in a Human Skeletal Muscle Microphysiological System - Main Analysis + S4

#### Anandita Ananthakumar, Yiling Liu, Cristina E. Fernandez, George A. Truskey, Deepak Voora

- Library Loading
- Read into data
- Statistical Analysis of Force and Immunoscoring Data
  - For Figure1
    - Figure 1B
      - Test Statistics
      - Generate Figure 1B
    - For Figure 1C
      - Test Statistics
      - Generate Figure 1C
  - For Figure2
    - Test Statistics
      - Test interaction terms: Donor.Type\*Statin.Concentration
      - Test main effects: Donor.Type, Statin.Concentration
    - Generate Figure 2
  - For Figure 4
    - For Figure 4A
      - Test Statistics
      - Generate Figure 4A
    - For Figure 4B
      - Test Statistics
      - Generate Figure 4B
- Statistical Analysis in Supplementary
  - For FigureS4
    - Test Statistics
    - Generate Figure S4

## Library Loading

The libraries required to run this analysis are:  
 limma,lme4, ggplot2 markdown

## Read into data

## Statistical Analysis of Force and Immunoscoring Data

### For Figure1

- Figure 1B: Control
- Figure 1C: Case

#### Figure 1B

##### Test Statistics

|  | Estimate | Std. Error | df | t value | Pr(>|t|) | Confidence Interval - 2.5% | Confidence Interval - 97.5% | LRT pvalue (Based on ML) |
| --- | --- | --- | --- | --- | --- | --- | --- | --- |
| Concentration | -0.154809434 | 0.0571690725256742 | 13.0000017595382 | -2.70792278343288 | 0.0179211289746616 | -0.270631035544108 | -0.038987829882056 | 0.0123352481583164 |

```
## <br>
```

```
##  Summary statistics
```

| AIC | R2m | R2c | Restricted log-likelihood |
| --- | --- | --- | --- |
| 9.24325 | 0.085605 | 0.6847969 | -0.621625 |

##### Generate Figure 1B

#### For Figure 1C

##### Test Statistics

|  | Estimate | Std. Error | df | t value | Pr(>|t|) | Confidence Interval - 2.5% | Confidence Interval - 97.5% | LRT pvalue (Based on ML) |
| --- | --- | --- | --- | --- | --- | --- | --- | --- |
| Concentration | -0.085988525 | 0.0395896866655679 | 9.00000136083708 | -2.17199306795187 | 0.0579251425323539 | -0.167270181408773 | -0.0047068638773939 | 0.0400794874713503 |

```
## <br>
```

```
##  Summary statistics
```

| AIC | R2m | R2c | Restricted log-likelihood |
| --- | --- | --- | --- |
| -3.685233 | 0.0467101 | 0.8118746 | 5.842617 |

##### Generate Figure 1C

### For Figure2

#### Test Statistics

##### Test interaction terms: Donor.Type\*Statin.Concentration

|  | Estimate | Std. Error | df | t value | Pr(>|t|) | Confidence Interval - 2.5% | Confidence Interval - 97.5% | LRT pvalue (Based on ML) |
| --- | --- | --- | --- | --- | --- | --- | --- | --- |
| Statin Concentration:Donor Type | 0.0607912868835739 | 0.0566645311400946 | 144.989940485669 | 1.07282784592846 | 0.285130722015859 | -0.0501121091524374 | 0.171953475581548 | 0.280203501320865 |

##### Test main effects: Donor.Type, Statin.Concentration

Since interaction term is not significant, only include model with main effects.

|  | Estimate | Std. Error | df | t value | Pr(>|t|) | Confidence Interval - 2.5% | Confidence Interval - 97.5% | LRT pvalue (Based on ML) |
| --- | --- | --- | --- | --- | --- | --- | --- | --- |
| Donor Type | -0.0157726 | 0.0865316 | 21.91194 | -0.1822757 | 0.8570422 | -0.1850171 | 0.1531641 | 0.8505890 |
| Statin Concentration | -0.1261391 | 0.0276690 | 145.97512 | -4.5588672 | 0.0000108 | -0.1803878 | -0.0715665 | 0.0000103 |

```
## <br>
```

```
##  Summary statistics for models only included main effects
```

| AIC | R2m | R2c | Restricted log-likelihood |
| --- | --- | --- | --- |
| -26.66496 | 0.0535908 | 0.5688313 | 18.33248 |

#### Generate Figure 2

### For Figure 4

#### For Figure 4A

##### Test Statistics

|  | Estimate | Std. Error | df | t value | Pr(>|t|) | Confidence Interval - 2.5% | Confidence Interval - 97.5% | LRT pvalue (Based on ML) |
| --- | --- | --- | --- | --- | --- | --- | --- | --- |
| Raw Tetanus Force | 0.159327018640533 | 0.0643277245937093 | 27.3652013338081 | 2.47680171569621 | 0.0197195550281466 | 0.0335099571143332 | 0.2907119504179 | 0.014530647431978 |

```
## <br>
```

```
##  Summary statistics
```

| AIC | R2m | R2c | Restricted log-likelihood |
| --- | --- | --- | --- |
| -54.2272 | 0.0996332 | 0.8081297 | 31.1136 |

##### Generate Figure 4A

#### For Figure 4B

##### Test Statistics

|  | Estimate | Std. Error | df | t value | Pr(>|t|) | Confidence Interval - 2.5% | Confidence Interval - 97.5% | LRT pvalue (Based on ML) |
| --- | --- | --- | --- | --- | --- | --- | --- | --- |
| Raw Tetanus Force | -6.78740840349728 | 3.467664179499 | 33.5841006017167 | -1.95734305635037 | 0.0586603314325203 | -13.5590515910523 | 0.0224468271064086 | 0.0507246039859171 |

```
## <br>
```

```
##  Summary statistics
```

| AIC | R2m | R2c | Restricted log-likelihood |
| --- | --- | --- | --- |
| 212.7766 | 0.1020345 | 0.4816512 | -102.3883 |

##### Generate Figure 4B

## Statistical Analysis in Supplementary

### For FigureS4

#### Test Statistics

|  | Estimate | Std. Error | df | t value | Pr(>|t|) | Confidence Interval - 2.5% | Confidence Interval - 97.5% | LRT pvalue (Based on ML) |
| --- | --- | --- | --- | --- | --- | --- | --- | --- |
| Statin Concentration | -0.0364385327589748 | 0.0176222099619033 | 16.3497362444032 | -2.06776180954317 | 0.0548668533854457 | -0.0715519498226711 | -0.000548846619433447 | 0.0469773442455232 |

```
## <br>
```

```
##  Summary statistics
```

| AIC | R2m | R2c | Restricted log-likelihood |
| --- | --- | --- | --- |
| -49.78499 | 0.0214066 | 0.8328399 | 28.8925 |

#### Generate Figure S4
