# Supplementary material for: Modeling statin myopathy in a human skeletal muscle microphysiological system
Source: PLoS One. 2020 Nov 25;15(11):e0242422. doi: 10.1371/journal.pone.0242422 (PMC7688150; doi:10.1371/journal.pone.0242422)
Supplement: S1 Data — (ZIP) [file pone.0242422.s001.zip › Data Repository/Scripts/codes_for_submission_S567.html]

Modeling Statin Myopathy in a Human Skeletal Muscle Microphysiological System - Supplentray S5, S6, S7


# Modeling Statin Myopathy in a Human Skeletal Muscle Microphysiological System - Supplentray S5, S6, S7

#### Anandita Ananthakumar, Yiling Liu, Cristina E. Fernandez, George A. Truskey, Deepak Voora

- Library Loading
- Read into data
- For FigureS5
  - For FigureS5A
    - Boxplot
    - Test Statistics
      - Model1: Twitch Kinetics:Time to Peak ~ statin concentration (ref:0) + age + gender(ref:Female) + (1|subject)
      - Model2: Twitch Kinetics:Time to Peak ~ Donor Type (ref:Control) + age + gender(ref:Female) + (1|subject)
      - Model3: Twitch Kinetics:Time to Peak ~ statin concentration (ref:0) + Donor Type (ref:Control) + age + gender(ref:Female) + (1|subject)
      - Model4 (Test interactions)
  - For FigureS5B
    - Boxplot
    - Test Statistics
      - Model1: Twitch Kinetics:Time to Half relaxation ~ statin concentration (ref:0) + age + gender(ref:Female) + (1|subject)
      - Model2: Twitch Kinetics:Time to Half relaxation ~ Donor Type (ref:Control) + age + gender(ref:Female) + (1|subject)
      - Model3: Twitch Kinetics:Time to Half relaxation ~ statin concentration (ref:0) + Donor Type (ref:Control) + age + gender(ref:Female) + (1|subject)
      - Model4 (Test interactions)
- For FigureS6
  - Boxplot
  - Test Statistics
    - Model1: Fatigue values:Time to Peak ~ statin concentration (ref:0) + age + gender(ref:Female) + (1|subject)
    - Model2: Fatigue values ~ Donor Type (ref:Control) + age + gender(ref:Female) + (1|subject)
    - Model3: Fatigue values ~ statin concentration (ref:0) + Donor Type (ref:Control) + age + gender(ref:Female) + (1|subject)
    - Model4 (Test interactions)
- For FigureS7
  - Boxplot
  - Test Statistics
    - Model1: Myoblast Purity ~ Donor Type (ref:Control) + age + gender(ref:Female)

## Library Loading

The libraries required to run this analysis are:  
 limma,lme4, ggplot2 markdown

## Read into data

## For FigureS5

### For FigureS5A

#### Boxplot

#### Test Statistics

##### Model1: Twitch Kinetics:Time to Peak ~ statin concentration (ref:0) + age + gender(ref:Female) + (1|subject)

|  | Estimate | Std. Error | df | t value | Pr(>|t|) | Confidence Interval - 2.5% | Confidence Interval - 97.5% | LRT pvalue (Based on ML) |
| --- | --- | --- | --- | --- | --- | --- | --- | --- |
| Statin Concentration | 38.409722 | 41.355812 | 23 | 0.9287624 | 0.3626577 | -44.22307 | 121.042516 | 0.3472079 |
| age | -9.045164 | 3.464224 | 21 | -2.6110218 | 0.0163171 | -15.65922 | -2.431107 | 0.0093886 |
| gender | -130.485577 | 64.539155 | 21 | -2.0218048 | 0.0561222 | -253.70673 | -7.264422 | 0.0388247 |

```
## <br>
```

```
##  Summary statistics
```

| AIC | R2m | R2c | Restricted log-likelihood |
| --- | --- | --- | --- |
| 603.3188 | 0.1995783 | 0.4371859 | -295.6594 |

##### Model2: Twitch Kinetics:Time to Peak ~ Donor Type (ref:Control) + age + gender(ref:Female) + (1|subject)

|  | Estimate | Std. Error | df | t value | Pr(>|t|) | Confidence Interval - 2.5% | Confidence Interval - 97.5% | LRT pvalue (Based on ML) |
| --- | --- | --- | --- | --- | --- | --- | --- | --- |
| Donor Type | -33.23133 | 59.136688 | 20 | -0.561941 | 0.5804000 | -143.41680 | 76.954145 | 0.5397664 |
| age | -9.26597 | 3.543928 | 20 | -2.614604 | 0.0165939 | -15.86914 | -2.662804 | 0.0078981 |
| gender | -125.53135 | 66.206602 | 20 | -1.896055 | 0.0724946 | -248.88972 | -2.172977 | 0.0463925 |

```
## <br>
```

```
##  Summary statistics
```

| AIC | R2m | R2c | Restricted log-likelihood |
| --- | --- | --- | --- |
| 603.1613 | 0.1939849 | 0.4474523 | -295.5807 |

##### Model3: Twitch Kinetics:Time to Peak ~ statin concentration (ref:0) + Donor Type (ref:Control) + age + gender(ref:Female) + (1|subject)

|  | Estimate | Std. Error | df | t value | Pr(>|t|) | Confidence Interval - 2.5% | Confidence Interval - 97.5% | LRT pvalue (Based on ML) |
| --- | --- | --- | --- | --- | --- | --- | --- | --- |
| Statin Concentration | 38.40972 | 41.355810 | 23 | 0.9287624 | 0.3626577 | -44.22239 | 121.041841 | 0.3472079 |
| Donor Type | -33.23133 | 59.136688 | 20 | -0.5619410 | 0.5804000 | -143.41663 | 76.953974 | 0.5397664 |
| age | -9.26597 | 3.543928 | 20 | -2.6146044 | 0.0165939 | -15.86913 | -2.662814 | 0.0078981 |
| gender | -125.53135 | 66.206602 | 20 | -1.8960549 | 0.0724946 | -248.88953 | -2.173169 | 0.0463925 |

```
## <br>
```

```
##  Summary statistics
```

| AIC | R2m | R2c | Restricted log-likelihood |
| --- | --- | --- | --- |
| 595.0168 | 0.2018049 | 0.4507466 | -290.5084 |

##### Model4 (Test interactions)

Full model: Twitch Kinetics:Time to Peak ~ statin concentration (ref:0) + Donor Type (ref:Control) + statin concentration (ref:0)\*Donor Type (ref:Control) + age + gender(ref:Female) + (1|subject)

Reduced model: Twitch Kinetics:Time to Peak ~ statin concentration (ref:0) + Donor Type (ref:Control) + age + gender(ref:Female) + (1|subject)

|  | Estimate | Std. Error | df | t value | Pr(>|t|) | Confidence Interval - 2.5% | Confidence Interval - 97.5% | LRT pvalue (Based on ML) |
| --- | --- | --- | --- | --- | --- | --- | --- | --- |
| Statin Concentration:Donor Type | 69.9547619017145 | 84.46351198403 | 21.9999999914721 | 0.828224641131916 | 0.416438167361151 | -95.0999905304365 | 235.009508941379 | 0.390660075511332 |

```
## <br>
```

```
##  Summary statistics of full model
```

| AIC | R2m | R2c | Restricted log-likelihood |
| --- | --- | --- | --- |
| 585.6225 | 0.2074574 | 0.4497076 | -284.8112 |

### For FigureS5B

#### Boxplot

#### Test Statistics

##### Model1: Twitch Kinetics:Time to Half relaxation ~ statin concentration (ref:0) + age + gender(ref:Female) + (1|subject)

|  | Estimate | Std. Error | df | t value | Pr(>|t|) | Confidence Interval - 2.5% | Confidence Interval - 97.5% | LRT pvalue (Based on ML) |
| --- | --- | --- | --- | --- | --- | --- | --- | --- |
| Statin Concentration | -37.680556 | 33.571660 | 22.99982 | -1.122392 | 0.2732768 | -104.2360008 | 28.874867 | 0.2579360 |
| age | 3.388584 | 2.234935 | 20.99992 | 1.516189 | 0.1443810 | -0.8784631 | 7.655631 | 0.1143441 |
| gender | 60.353632 | 41.637270 | 20.99992 | 1.449510 | 0.1619660 | -19.1422700 | 139.849536 | 0.1303308 |

```
## <br>
```

```
##  Summary statistics
```

| AIC | R2m | R2c | Restricted log-likelihood |
| --- | --- | --- | --- |
| 575.3187 | 0.0933358 | 0.1623978 | -281.6594 |

##### Model2: Twitch Kinetics:Time to Half relaxation ~ Donor Type (ref:Control) + age + gender(ref:Female) + (1|subject)

|  | Estimate | Std. Error | df | t value | Pr(>|t|) | Confidence Interval - 2.5% | Confidence Interval - 97.5% | LRT pvalue (Based on ML) |
| --- | --- | --- | --- | --- | --- | --- | --- | --- |
| Donor Type | -26.709059 | 37.985269 | 19.99997 | -0.7031425 | 0.4900719 | -96.3351503 | 42.91703 | 0.4445438 |
| age | 3.211116 | 2.276371 | 19.99997 | 1.4106290 | 0.1737191 | -0.9614188 | 7.38365 | 0.1314885 |
| gender | 64.335498 | 42.526487 | 19.99997 | 1.5128336 | 0.1459642 | -13.6145367 | 142.28553 | 0.1069480 |

```
## <br>
```

```
##  Summary statistics
```

| AIC | R2m | R2c | Restricted log-likelihood |
| --- | --- | --- | --- |
| 575.8419 | 0.0810268 | 0.157239 | -281.9209 |

##### Model3: Twitch Kinetics:Time to Half relaxation ~ statin concentration (ref:0) + Donor Type (ref:Control) + age + gender(ref:Female) + (1|subject)

|  | Estimate | Std. Error | df | t value | Pr(>|t|) | Confidence Interval - 2.5% | Confidence Interval - 97.5% | LRT pvalue (Based on ML) |
| --- | --- | --- | --- | --- | --- | --- | --- | --- |
| Statin Concentration | -37.680556 | 33.571626 | 22.99999 | -1.1223929 | 0.2732762 | -103.951089 | 28.589984 | 0.2576480 |
| Donor Type | -26.709059 | 37.985293 | 20.00000 | -0.7031421 | 0.4900721 | -97.484431 | 44.066314 | 0.4439405 |
| age | 3.211116 | 2.276373 | 20.00000 | 1.4106282 | 0.1737194 | -1.030293 | 7.452524 | 0.1313571 |
| gender | 64.335498 | 42.526513 | 20.00000 | 1.5128327 | 0.1459644 | -14.901216 | 143.572214 | 0.1068434 |

```
## <br>
```

```
##  Summary statistics
```

| AIC | R2m | R2c | Restricted log-likelihood |
| --- | --- | --- | --- |
| 567.7182 | 0.1015625 | 0.1808732 | -276.8591 |

##### Model4 (Test interactions)

Full model: Twitch Kinetics:Time to Half relaxation ~ statin concentration (ref:0) + Donor Type (ref:Control) + statin concentration (ref:0)\*Donor Type (ref:Control) + age + gender(ref:Female) + (1|subject)

Reduced model: Twitch Kinetics:Time to Half relaxation ~ statin concentration (ref:0) + Donor Type (ref:Control) + age + gender(ref:Female) + (1|subject)

|  | Estimate | Std. Error | df | t value | Pr(>|t|) | Confidence Interval - 2.5% | Confidence Interval - 97.5% | LRT pvalue (Based on ML) |
| --- | --- | --- | --- | --- | --- | --- | --- | --- |
| Statin Concentration:Donor Type | 104.138095238571 | 65.9913086323826 | 21.9999995532054 | 1.57805773815902 | 0.1288241661426 | -24.768157712664 | 233.044804495641 | 0.108660270628218 |

```
## <br>
```

```
##  Summary statistics for the full model
```

| AIC | R2m | R2c | Restricted log-likelihood |
| --- | --- | --- | --- |
| 557.0573 | 0.1401035 | 0.2427118 | -270.5287 |

## For FigureS6

### Boxplot

### Test Statistics

#### Model1: Fatigue values:Time to Peak ~ statin concentration (ref:0) + age + gender(ref:Female) + (1|subject)

|  | Estimate | Std. Error | df | t value | Pr(>|t|) | Confidence Interval - 2.5% | Confidence Interval - 97.5% | LRT pvalue (Based on ML) |
| --- | --- | --- | --- | --- | --- | --- | --- | --- |
| Statin Concentration | -0.9276642 | 3.5282602 | 22.99975 | -0.2629240 | 0.7949501 | -7.7326674 | 5.877339 | 0.7852265 |
| age | 0.1118308 | 0.2209486 | 20.99978 | 0.5061396 | 0.6180310 | -0.3079044 | 0.531566 | 0.5947015 |
| gender | 4.4399079 | 4.1163139 | 20.99978 | 1.0786126 | 0.2929945 | -3.3798390 | 12.259655 | 0.2593386 |

```
## <br>
```

```
##  Summary statistics
```

| AIC | R2m | R2c | Restricted log-likelihood |
| --- | --- | --- | --- |
| 374.4968 | 0.0267324 | 0.0414851 | -181.2484 |

#### Model2: Fatigue values ~ Donor Type (ref:Control) + age + gender(ref:Female) + (1|subject)

|  | Estimate | Std. Error | df | t value | Pr(>|t|) | Confidence Interval - 2.5% | Confidence Interval - 97.5% | LRT pvalue (Based on ML) |
| --- | --- | --- | --- | --- | --- | --- | --- | --- |
| Donor Type | 1.2129424 | 3.7917096 | 19.99981 | 0.3198933 | 0.7523670 | -5.8312901 | 8.2571749 | 0.7307420 |
| age | 0.1198902 | 0.2272286 | 19.99980 | 0.5276194 | 0.6035684 | -0.3022547 | 0.5420351 | 0.5707085 |
| gender | 4.2590789 | 4.2450163 | 19.99980 | 1.0033127 | 0.3276954 | -3.6273062 | 12.1454640 | 0.2830263 |

```
## <br>
```

```
##  Summary statistics
```

| AIC | R2m | R2c | Restricted log-likelihood |
| --- | --- | --- | --- |
| 374.3208 | 0.0274516 | 0.0826126 | -181.1604 |

#### Model3: Fatigue values ~ statin concentration (ref:0) + Donor Type (ref:Control) + age + gender(ref:Female) + (1|subject)

|  | Estimate | Std. Error | df | t value | Pr(>|t|) | Confidence Interval - 2.5% | Confidence Interval - 97.5% | LRT pvalue (Based on ML) |
| --- | --- | --- | --- | --- | --- | --- | --- | --- |
| Statin Concentration | -0.9276642 | 3.5282613 | 22.99980 | -0.2629239 | 0.7949502 | -7.7242599 | 5.8689315 | 0.7849677 |
| Donor Type | 1.2129424 | 3.7917137 | 19.99985 | 0.3198929 | 0.7523673 | -5.8774012 | 8.3032881 | 0.7305415 |
| age | 0.1198902 | 0.2272288 | 19.99985 | 0.5276188 | 0.6035688 | -0.3050181 | 0.5447986 | 0.5704107 |
| gender | 4.2590789 | 4.2450210 | 19.99985 | 1.0033116 | 0.3276959 | -3.6789301 | 12.1970902 | 0.2826575 |

```
## <br>
```

```
##  Summary statistics
```

| AIC | R2m | R2c | Restricted log-likelihood |
| --- | --- | --- | --- |
| 371.9107 | 0.028292 | 0.0642304 | -178.9554 |

#### Model4 (Test interactions)

Full model: Fatigue values ~ statin concentration (ref:0) + Donor Type (ref:Control) + statin concentration (ref:0)\*Donor Type (ref:Control) + age + gender(ref:Female) + (1|subject)

Reduced model: Fatigue values ~ statin concentration (ref:0) + Donor Type (ref:Control) + age + gender(ref:Female) + (1|subject)

|  | Estimate | Std. Error | df | t value | Pr(>|t|) | Confidence Interval - 2.5% | Confidence Interval - 97.5% | LRT pvalue (Based on ML) |
| --- | --- | --- | --- | --- | --- | --- | --- | --- |
| Statin Concentration:Donor Type | 14.6963784298569 | 6.61269728311203 | 21.9999998648991 | 2.22244838991641 | 0.0368451697776336 | 1.78339545617625 | 27.6093995795257 | 0.0270601801038735 |

```
## <br>
```

```
##  Summary statistics
```

| AIC | R2m | R2c | Restricted log-likelihood |
| --- | --- | --- | --- |
| 363.6588 | 0.1105346 | 0.2132949 | -173.8294 |

## For FigureS7

### Boxplot

### Test Statistics

#### Model1: Myoblast Purity ~ Donor Type (ref:Control) + age + gender(ref:Female)

```
##  Summary statistics
```

|  | Estimate | Std. Error | t value | Pr(>|t|) | Confidence Interval - 2.5% | Confidence Interval - 97.5% |
| --- | --- | --- | --- | --- | --- | --- |
| Donor Type | -8.325616 | 7.6545401 | -1.0876703 | 0.2896752 | -24.292707 | 7.6414750 |
| age | -0.195826 | 0.4587193 | -0.4268973 | 0.6740144 | -1.152698 | 0.7610456 |
| gender | -9.290428 | 8.5696562 | -1.0841074 | 0.2912136 | -27.166418 | 8.5855616 |

```
## Residual standard error: 18.1046263043 on 20 degrees of freedom
```

```
## <br>
```

```
##  Summary statistics
```

| AIC | R square |
| --- | --- |
| 212.7494 | 0.1198485 |
