# Supplementary material for: Modeling statin myopathy in a human skeletal muscle microphysiological system
Source: PLoS One. 2020 Nov 25;15(11):e0242422. doi: 10.1371/journal.pone.0242422 (PMC7688150; doi:10.1371/journal.pone.0242422)
Supplement: S1 Table — (DOCX) [file pone.0242422.s002.docx]

**Donor Diagnostic Information**

| **S1 Table. Diagnosis Codes Used to Search for Potential Cases** | | | |
| --- | --- | --- | --- |
| **Code** | **Code Set** | **Description** | |
| 359.4 | ICD-9-CM | Toxic Myopathy |  |
| 359.9 | ICD-9-CM | Myopathy, Unspecified |  |
| 728.88 | ICD-9-CM | Rhabdomyolysis |  |
| 729.1 | ICD-9-CM | Myalgia and Myositis, Unspecified |  |
| 790.5 | ICD-9-CM | Other Nonspecific Abnormal Serum Enzyme Levels |  |
| E942.2 | ICD-9-CM | Antilipemic And Antiarteriosclerotic Drugs Causing Adverse Effect in Therapeutic Use |  |
| G72.0 | ICD-10-CM | Drug-Induced Myopathy |  |
| G72.9 | ICD-10-CM | Myopathy, Unspecified |  |
| M62.82 | ICD-10-CM | Rhabdomyolysis |  |
| M79.1 | ICD-10-CM | Myalgia |  |
| R74.8 | ICD-10-CM | Abnormal Levels of Other Serum Enzymes |  |
| T46.6X1A | ICD-10-CM | Poisoning by Antihyperlipidemic And Antiarteriosclerotic Drugs, Accidental (Unintentional), Initial Encounter |  |
