# Supplementary material for: Modeling statin myopathy in a human skeletal muscle microphysiological system
Source: PLoS One. 2020 Nov 25;15(11):e0242422. doi: 10.1371/journal.pone.0242422 (PMC7688150; doi:10.1371/journal.pone.0242422)
Supplement: S2 Table — (DOCX) [file pone.0242422.s003.docx]

**Donor Diagnostic Information**

| **S2 Table. Problem Names used to Search Problem Lists to Identify Cases** |
| --- |
| Adverse Reaction To Statin Medication (6) |
| Allergy To Statin Medication (25) |
| HMG-COA Myositis (3) |
| Myalgia (2558) |
| Myalgia And Myositis (424) |
| Myalgia And Myositis, Unspecified (422) |
| Refusal Of Statin Medication By Patient (149) |
| Rhabdomyolysis Due To Statin Therapy (7) |
| Simvastatin-Induced Rhabdomyolysis (5) |
| Statin Intolerance (1838) |
| Statin Myopathy (51) |
| Statin-Induced Myositis (15) |
