# Supplementary material for: Modeling statin myopathy in a human skeletal muscle microphysiological system
Source: PLoS One. 2020 Nov 25;15(11):e0242422. doi: 10.1371/journal.pone.0242422 (PMC7688150; doi:10.1371/journal.pone.0242422)
Supplement: S3 Table — (DOCX) [file pone.0242422.s004.docx]

**Donor Characteristics**

| **S3 Table. Baseline Donor Demographics** | | | | | | | |
| --- | --- | --- | --- | --- | --- | --- | --- |
|  |  | Case | | Control | | Totals | |
|  |  | N | % | N | % | N | % |
| Totals |  | 20 | 47.6 | 22.0 | 52.4 | 42 | 100.0 |
| Sex | Male | 16 | 80.0 | 16.0 | 72.7 | 32 | 76.2 |
| Alcohol | Yes | 10 | 50.0 | 10.0 | 45.5 | 20 | 47.6 |
| Alcohol Use at Time of Myopathy | Yes | 15 | 75.0 | NA | NA | 15 | 35.7 |
| Tobacco Use at Time of Myopathy | Yes | 5 | 25.0 | NA | NA | 5 | 11.9 |
| Ethnicity | Not Hispanic or Latino | 18 | 90.0 | 22.0 | 100.0 | 40 | 95.2 |
| Race | white | 10 | 50.0 | 18.0 | 81.8 | 28 | 66.7 |

|  |  |  |  |  |  |  |
| --- | --- | --- | --- | --- | --- | --- |
| Age, years (mean, SD) | 62.9 | 8.3 | 64.2 | 7.1 | 63.6 | 7.6 |
| Height, inches (mean, SD) | 69.6 | 4.5 | 68.7 | 3.5 | 69.1 | 4.0 |
| Weight, lbs (mean, SD) | 214.3 | 50.9 | 217.0 | 50.7 | 215.7 | 50.2 |
