# Supplementary material for: Modeling statin myopathy in a human skeletal muscle microphysiological system
Source: PLoS One. 2020 Nov 25;15(11):e0242422. doi: 10.1371/journal.pone.0242422 (PMC7688150; doi:10.1371/journal.pone.0242422)
Supplement: S4 Table — (DOCX) [file pone.0242422.s005.docx]

**Donor Characteristics**

| **S4 Table. Baseline Comorbidities and Concomitant Medications** | | | | | | | |
| --- | --- | --- | --- | --- | --- | --- | --- |
|  |  | Case | | Control | | Totals | |
|  |  | N | % | N | % | N | % |
|  | Totals | 20 | 47.6 | 22.0 | 52.4 | 42 | 100 |
| Coronary Artery | Yes | 4 | 20 | 14 | 63.6 | 18 | 42.9 |
| Diabetes | Yes | 8 | 40 | 8 | 36.4 | 16 | 38.1 |
| Cerebrovascular disease | Yes | 0 | 0 | 3 | 13.6 | 3 | 7.1 |
| Hyperlipidemia | Yes | 19 | 95 | 20 | 90.9 | 39 | 92.9 |
| Hypertension | Yes | 15 | 75 | 16 | 72.7 | 31 | 73.8 |
| Congestive Heart Failure |  | 0 | 0 | 0 | 0 | 0 | 0 |
| Osteoarthritis | Yes | 6 | 30 | 2 | 9.1 | 8 | 19.0 |
| Rheumatoid Arthritis |  | 0 | 0 | 0 | 0 | 0 | 0 |
| Obesity | Yes | 6 | 30 | 8 | 36.4 | 14 | 33.3 |
| Hypothyroidism | Yes | 1 | 5 | 1 | 4.5 | 2 | 4.8 |
| Concomitant Medications | | | | | | | |
|  | Anti-inflammatory agents | 5 | 25 | 7 | 31.8 | 12 | 28.6 |
|  | Analgesics  non-opioid | 5 | 25 | 13 | 59.1 | 18 | 42.9 |
|  | Analgesics - opioid | 4 | 20 | 4 | 18.2 | 8 | 19.0 |
|  | Non DHP calcium channel blocker | 4 | 20 | 11 | 50.0 | 15 | 35.7 |
|  | Synthroid | 2 | 10 | 3 | 13.6 | 5 | 11.9 |
|  | Fibrates | 2 | 10 | 4 | 18.2 | 6 | 14.3 |
