# Supplementary material for: Modeling statin myopathy in a human skeletal muscle microphysiological system
Source: PLoS One. 2020 Nov 25;15(11):e0242422. doi: 10.1371/journal.pone.0242422 (PMC7688150; doi:10.1371/journal.pone.0242422)
Supplement: S5 Table — (DOCX) [file pone.0242422.s006.docx]

**Donor Characteristics**

| **S5 Table: Myopathy Symptoms in Cases** | | |
| --- | --- | --- |
|  |  | N |
| Myopathy Characteristics | Pain | 28 |
|  | Weakness | 13 |
|  | Stiffness | 8 |
|  | Rhabdomyolysis | 0 |
|  | Asymptomatic (i.e. CK elevation only) | 0 |
|  | Other | 5 |
|  |  |  |
| Statin most likely attributable to symptoms | Simvastatin (Zocor, Vytorin, Simcor) | 6 |
|  | Atorvastatin (Lipitor) | 8 |
|  | Rosuvastatin (Crestor) | 3 |
|  | Pravastatin (Pravachol) | 2 |
|  | Lovastatin (Altoprev, Mevocor) | 1 |
| *Values sum to greater than the total number of cases because participants may have reported multiple different episodes of myopathy | | |
