# Supplementary material for: Modeling statin myopathy in a human skeletal muscle microphysiological system
Source: PLoS One. 2020 Nov 25;15(11):e0242422. doi: 10.1371/journal.pone.0242422 (PMC7688150; doi:10.1371/journal.pone.0242422)
Supplement: S6 Table — (DOCX) [file pone.0242422.s007.docx]

**Donor Characteristics**

| **S6 Table. Patient-Reported Symptoms: Answer 1 in Any of the Questions in Muscle Weakness, Muscle Pain, Muscle Stiffness** | | |
| --- | --- | --- |
|  | Case | Control |
| Yes | 14 | 8 |
| No | 7 | 13 |
