# Supplementary material for: Modeling statin myopathy in a human skeletal muscle microphysiological system
Source: PLoS One. 2020 Nov 25;15(11):e0242422. doi: 10.1371/journal.pone.0242422 (PMC7688150; doi:10.1371/journal.pone.0242422)
Supplement: S7 Table — (DOCX) [file pone.0242422.s008.docx]

**S7 Table:** **Statin Type, Gender, Age and Creatinine Kinase Level of Donors Tested.**

| **Experiment** | **Type of Statin** | **Control** | **Age** | **Case (Myopathy)** | **Age** | **Gender** | **Creatinine Kinase Level (U/L)** |
| --- | --- | --- | --- | --- | --- | --- | --- |
| 1 | Simvastatin | 105 | 77 | 140 | 74 | Male | 821 |
| 2 | Simvastatin | 116 | 61 | 119 | 61 | Female | 621 |
| 3 | Atorvastatin | 102 | 60 | 139 | 62 | Male | 716 |
| 4 | Atorvastatin | 136 | 61 | 117 | 65 | Male | 915 |
| 5 | Atorvastatin | 128 | 52 | 143 | 60 | Male | 599 |
| 6 | Atorvastatin | 130 | 69 | 129 | 70 | Male | 427 |
| 7 | Rosuvastatin | 111 | 51 | 138 | 45 | Male | N/A |
| 8 | Pravastatin | 110 | 63 | 132 | 45 | Male | 384 |
| 9 | Pravastatin | 142 | 66 | 123 | 64 | Female | 357 |
| 10 | Lovastatin | 141 | 64 | 125 | 70 | Male | 474 |
| 11 | Atorvastatin | 135 | 73 | - |  | Female | - |
| 12 | Simvastatin | 107 | 74 | - |  | Female | - |
| 13 | Atorvastatin | 113 | 71 | - |  | Female | - |
| 14 | Atorvastatin | 131 | 59 | - |  | Male | - |
| Mean |  |  | 64.4 |  | 61.6 |  |  |
| Standard Deviation |  |  | 7.8 |  | 9.8 |  |  |
