# Supplementary material for: Modeling statin myopathy in a human skeletal muscle microphysiological system
Source: PLoS One. 2020 Nov 25;15(11):e0242422. doi: 10.1371/journal.pone.0242422 (PMC7688150; doi:10.1371/journal.pone.0242422)
Supplement: S8 Table — (DOCX) [file pone.0242422.s009.docx]

**Report of Statistical Data**

**S8 Table:** Details of statistical data including parameter, estimate, standard error, degree of freedom, t value, p-value, confidence interval.

| **Figure** | **Parameter** | **Estimate** | **Standard Error** | **Degree of Freedom** | **t value** | **Pr(>\|t\|)** | **Confidence Interval - 2.5%** | **Confidence Interval - 97.5%** | **LRT P-Value (Based on ML)** |
| --- | --- | --- | --- | --- | --- | --- | --- | --- | --- |
| Figure 1 B | Concentration | -0.155 | 0.057 | 13.000 | -2.708 | 0.018 | -0.271 | -0.039 | 0.012 |
| Figure 1 C | Concentration | -0.086 | 0.040 | 9.000 | -2.172 | 0.058 | -0.167 | -0.005 | 0.040 |
| Figure 2 | Statin Concentration: Donor Type | 0.061 | 0.057 | 144.990 | 1.073 | 0.285 | -0.050 | 0.172 | 0.280 |
| Figure 2 | Donor Type | -0.016 | 0.087 | 21.912 | -0.182 | 0.857 | -0.185 | 0.153 | 0.851 |
| Figure 2 | Statin Concentration | -0.126 | 0.028 | 145.975 | -4.559 | 0.0000108 | -0.180 | -0.072 | 0.0000103 |
| Figure 4 A | Raw Tetanus Force | 0.159 | 0.064 | 27.365 | 2.477 | 0.0197 | 0.033 | 0.291 | 0.0145 |
| Figure 4 B | Raw Tetanus Force | -6.787 | 3.468 | 33.584 | -1.957 | 0.059 | -13.559 | 0.022 | 0.051 |
| Figure S4 | Statin Concentration | -0.0364 | 0.0176 | 16.350 | -2.068 | 0.0549 | -0.072 | -0.00055 | 0.047 |
| Figure S5A | Statin Concentration: Donor Type | 69.955 | 84.464 | 22.000 | 0.828 | 0.416 | -95.010 | 235.010 | 0.391 |
| Figure S5B | Statin Concentration: Donor Type | 104.138 | 65.991 | 22.000 | 1.578 | 0.129 | -24.768 | 233.045 | 0.109 |
| Figure S6 | Statin Concentration: Donor Type | 14.696 | 6.613 | 22.000 | 2.222 | 0.037 | 1.783 | 27.609 | 0.027 |
| Figure S7 | Donor Type | -8.326 | 7.654 | 20.000 | -1.088 | 0.289 | -24.292 | 7.641 |  |
| Figure S7 | Age | -0.196 | 0.459 | 20.000 | -0.427 | 0.674 | -1.152 | 0.761 |  |
| Figure S7 | Gender | -9.290 | 8.569 | 20.000 | -1.084 | 0.291 | -27.166 | 8.585 |  |
