# Supplementary material for: Modeling statin myopathy in a human skeletal muscle microphysiological system
Source: PLoS One. 2020 Nov 25;15(11):e0242422. doi: 10.1371/journal.pone.0242422 (PMC7688150; doi:10.1371/journal.pone.0242422)
Supplement: S9 Table — (DOCX) [file pone.0242422.s010.docx]

**Report of Statistical Data**

| **Figure** | **AIC** | **R^2^ M** | **R^2^ C** | **Restricted Log Likelihood** |
| --- | --- | --- | --- | --- |
| Figure 1 B | 9.243 | 0.086 | 0.685 | -0.622 |
| Figure 1 C | -3.685 | 0.047 | 0.812 | 5.843 |
| Figure 2 | -26.664 | 0.054 | 0.569 | 18.332 |
| Figure 4 A | -54.227 | 0.100 | 0.808 | 31.114 |
| Figure 4 B | 212.777 | 0.102 | 0.482 | -102.388 |
| Figure S4 | -49.785 | 0.021 | 0.833 | 28.893 |
| Figure S5A | 585.623 | 0.207 | 0.450 | -284.811 |
| Figure S5B | 557.057 | 0.140 | 0.243 | -270.529 |
| Figure S6 | 363.658 | 0.111 | 0.213 | -173.829 |
| Figure S7 | 212.749 |  | 0.120 (Multiple R^2^) |  |

**S9 Table:** Details of statistical data including AIC, RM, RC and Restricted Log Likelihood.
