# Supplementary figures and images for: Modeling statin myopathy in a human skeletal muscle microphysiological system
Source: PLoS One. 2020 Nov 25;15(11):e0242422. doi: 10.1371/journal.pone.0242422 (PMC7688150; doi:10.1371/journal.pone.0242422)

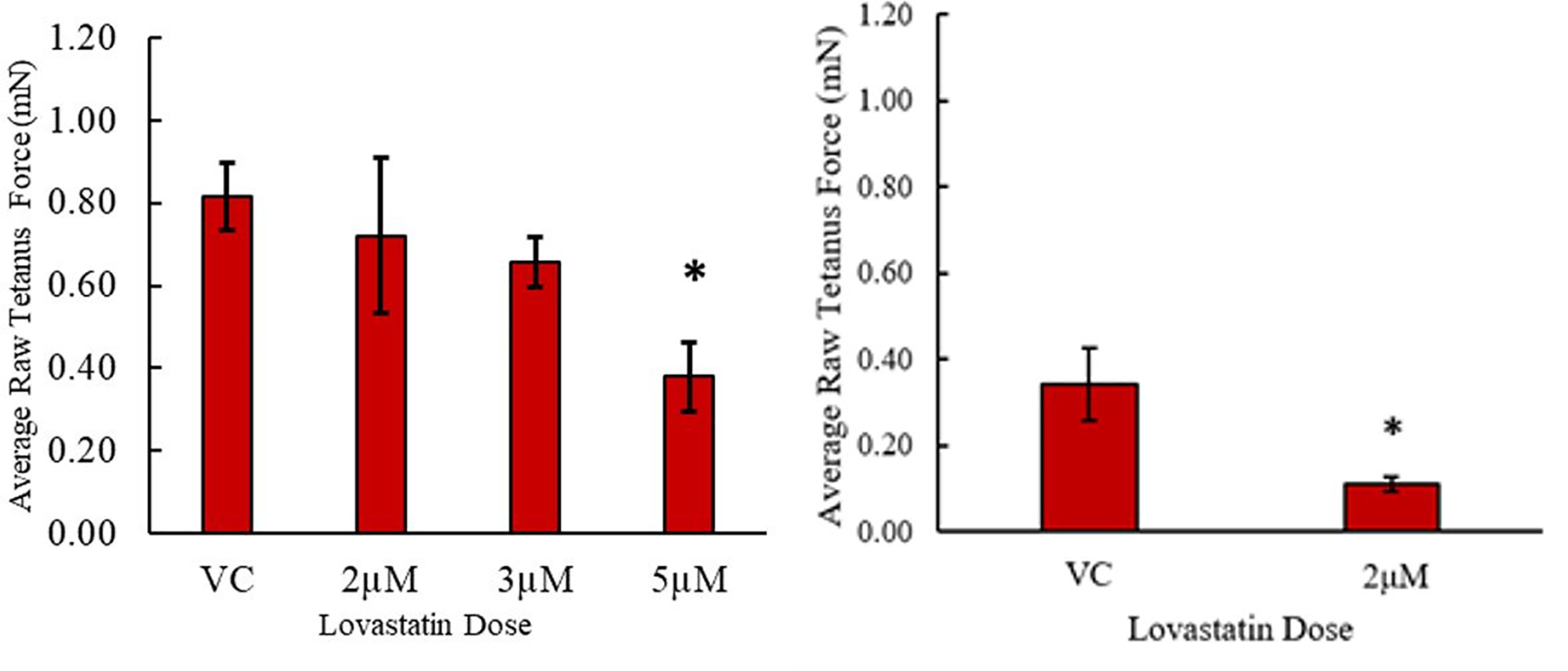

Supplement: S1 Fig — Myobundles made with myoblasts from healthy donors received (A) 2, 3 and 5 μM of lovastatin for 5 days (B) 2 μM of lovastatin for 10 days. There is a significant difference between conditions with respect to their respective vehicle controls. * p < 0.05. A ~58% reduction in force is seen between VC at day 5 and Day 10. Data reported as mean ±SEM, n = 3–4 biological replicates per condition. (TIF) [file pone.0242422.s011.tif]

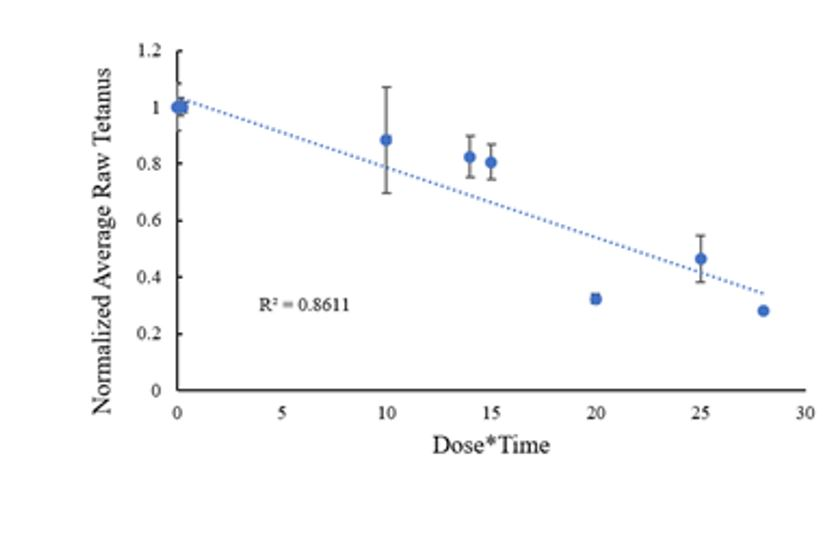

Supplement: S2 Fig — (TIF) [file pone.0242422.s012.tif]

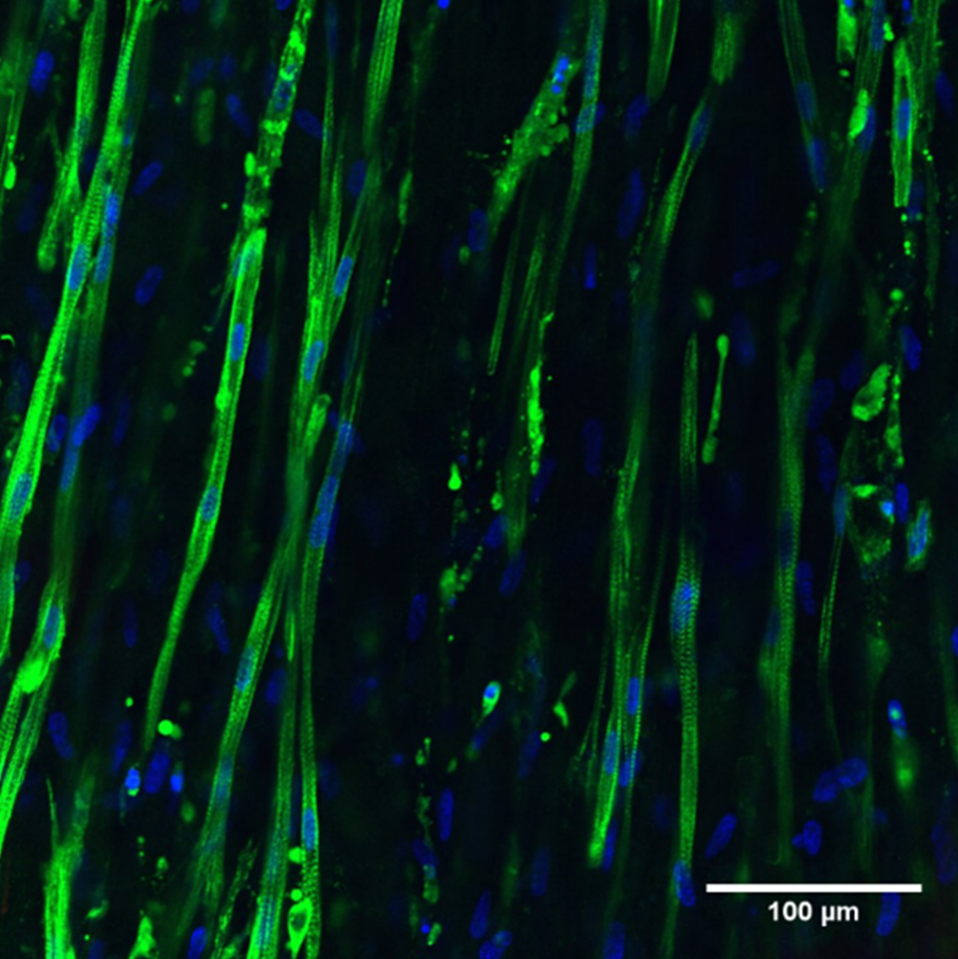

Supplement: S3 Fig — (TIF) [file pone.0242422.s013.tif]

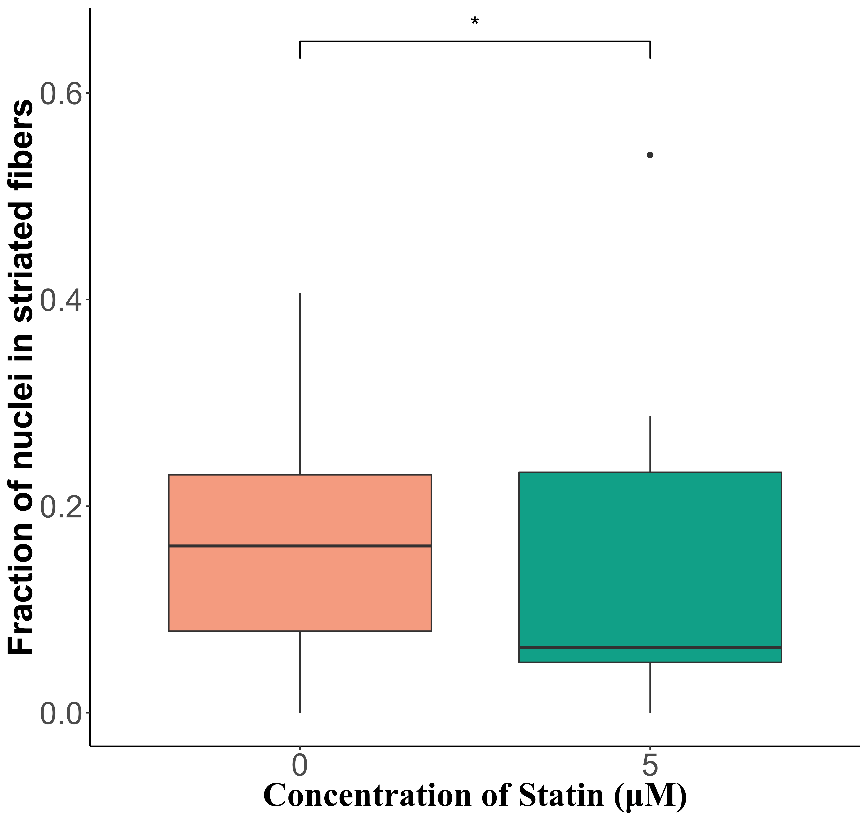

Supplement: S4 Fig — P<0.05. (TIF) [file pone.0242422.s014.tif]

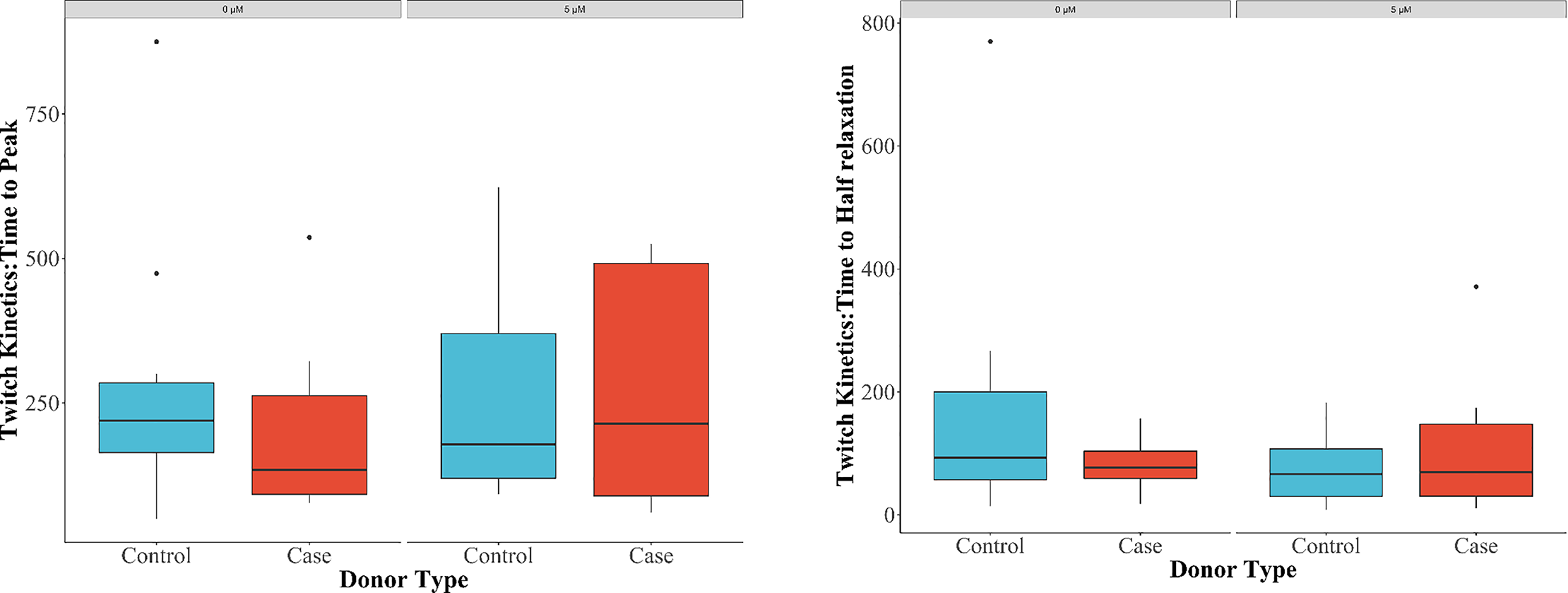

Supplement: S5 Fig — A, B. There is no significant differences in Twitch Kinetics between case and control. (TIF) [file pone.0242422.s015.tif]

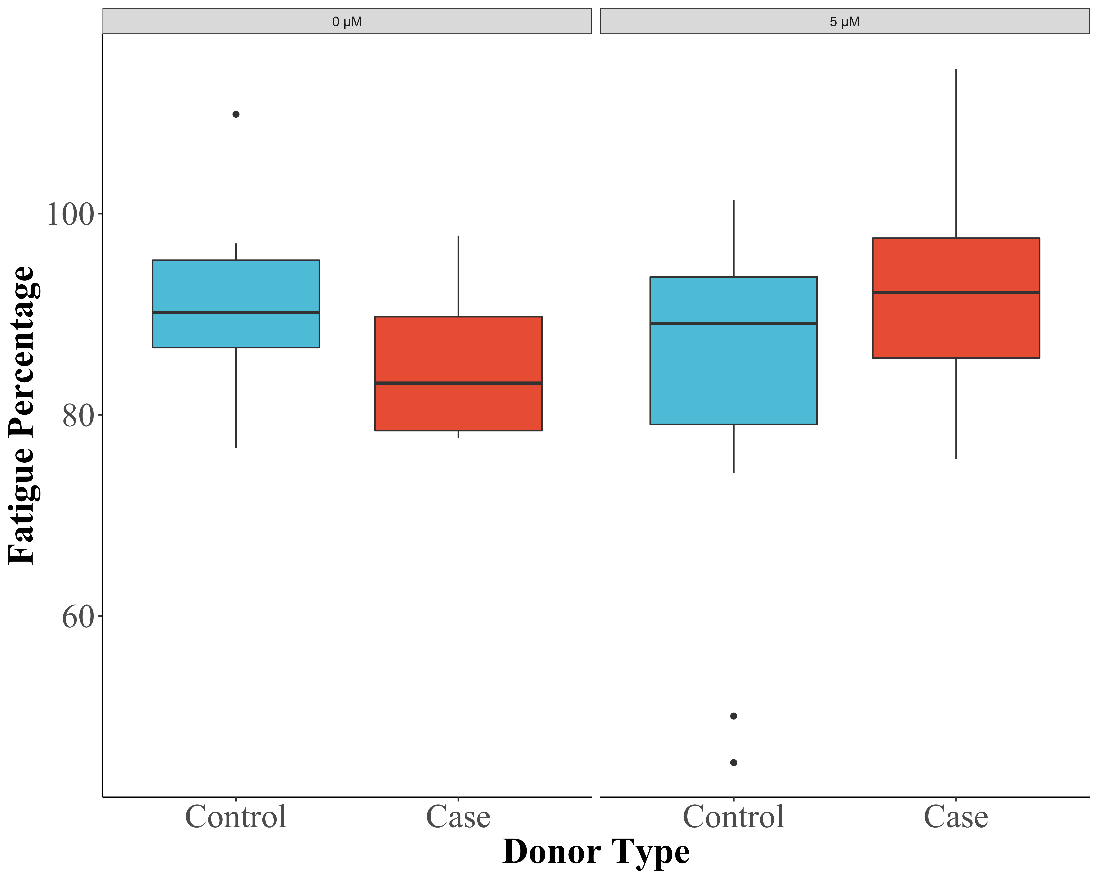

Supplement: S6 Fig — (TIF) [file pone.0242422.s016.tif]

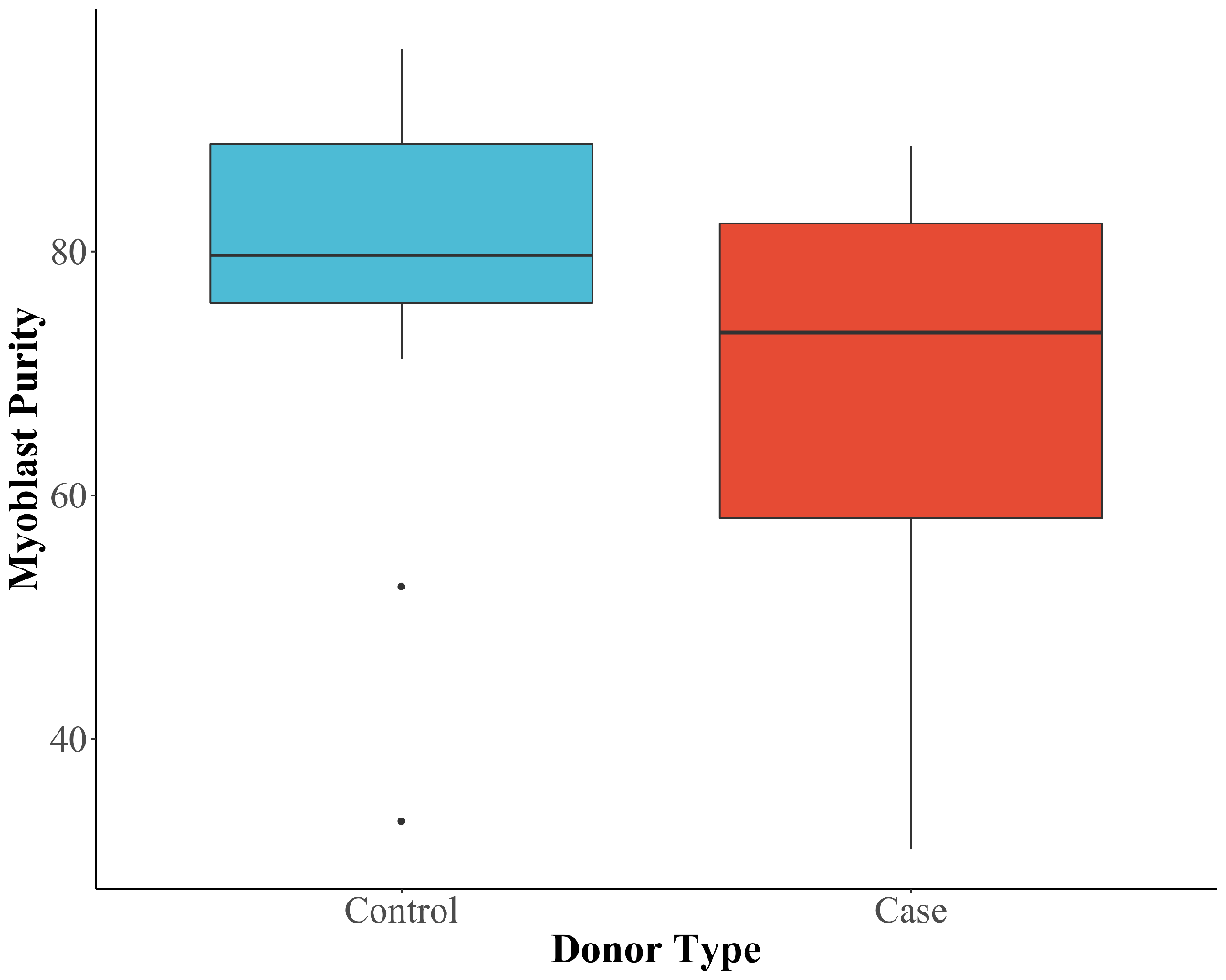

Supplement: S7 Fig — (TIF) [file pone.0242422.s017.tif]
